# A sensitivity analysis of preprocessing pipelines: Toward a solution for multiverse analyses

**DOI:** 10.1162/imag_a_00523

**Published:** 2025-04-28

**Authors:** Brice Ozenne, Martin Nørgaard, Cyril Pernet, Melanie Ganz

**Affiliations:** Neurobiology Research Unit, Rigshospitalet Blegdamsvej, Copenhagen, Denmark; Section of Biostatistics, University of Copenhagen, Copenhagen, Denmark; Department of Computer Science, University of Copenhagen, Copenhagen, Denmark; Molecular Imaging Branch, National Institute of Mental Health (NIMH), Bethesda, MD, United States

**Keywords:** neuroimaging, preprocessing and multiverse analyses

## Abstract

Being able to aggregate results from many acceptable data analysis pipelines (multiverse analyses) is a desirable feature in almost all aspects of imaging neuroscience. This is because multiple noise sources may contaminate the acquired imaging data, and different pipelines will attenuate or remove those noise source effects differentially. Here, we used multiple preprocessing pipelines that are known to impact the final results and conclusions of Positron Emission Tomography (PET) neuroimaging studies significantly. We developed conceptual and practical tools for statistical analyses that aggregate pipeline results and a new sensitivity analysis testing for hypotheses across pipelines, such as “no effect across all pipelines” or “at least one pipeline with no effect”. The proposed framework is generic and can be applied to any multiverse scenario. Code to reproduce all analyses and figures is openly available, including a step-by-step tutorial, so other researchers can carry out their own multiverse analysis.

## 1 Introduction

Modern neuroimaging techniques have provided unique opportunities to measure complex signaling pathways in the living human brain with the goal of identifying reliable biomarkers of disease states and treatment outcomes (Poldrack et al., 2020). Data arising from state-of-the-art neuroimaging techniques are, however, often contaminated with noise confounds such as motion-related artefacts, affecting both the spatial and temporal correlation structure of the data (Nørgaard, Ozenne, et al., 2019). Carefully designed preprocessing steps have been developed to remove unwanted noise sources, but in the absence of a “ground truth” it remains a major challenge to evaluate the impact of preprocessing choices on subsequent statistical analyses and results. Over time, preprocessing pipelines (i.e., a set of preprocessing steps) have become more complex and flexible, and this increase in researcher degrees of freedom (termed multiverse analyses) has consistently been shown to affect the outcomes of neuroimaging studies (Botvinik-Nezer et al., 2020;Carp, 2012;Nørgaard, Ganz, et al., 2019). The most common approach in the neuroimaging field is, to date, to use a single pipeline and not consider the heterogeneity of preprocessing choices. This approach not only makes abstraction of the multitude of possible results more difficult, but is likely also sub-optimal because the best pipeline is more often than not, study, population, or even subject dependent (Churchill et al., 2015;Nørgaard, Ganz, et al., 2019). More concerning, neuroscientists might be tempted to “tune” the pipeline in order to obtain the most satisfying results. This will generally lead to spurious and non-reproducible results since the variability induced by the choice of pipeline is now conditioned on the desired outcome. However, since it is neither realistic nor optimal to move toward a single unified preprocessing pipeline, there is an urgent need for a statistical framework allowing to explore results among many preprocessing pipelines in a principled way.

The aim of this work is, thus, to provide a statistical framework that can aggregate the evidence from multiverse analyses to produce conclusions robust to the choice of the pipeline. More specifically, the present paper proposes a statistical sensitivity analysis providing:

(i) visualizations of the heterogeneity of several preprocessing pipelines
(ii) estimation of a global effect across all preprocessing pipelines
(iii) quantification of the proportion of pipelines with evidence for an effect
(iv) a statistical framework for testing hypotheses across pipelines such as “no effect across all pipelines”, “at least one pipeline with no effect”

In this work, as an exemplary case, we use Positron Emission Tomography (PET) neuroimaging data to carry out a real-world multiverse analysis. However, our framework is generic and may be used in any multiverse scenario. The corresponding software is available as an R package called LMMstar freely accessible on CRAN, and our Github repository contains the code and data to reproduce all simulations and figures. Finally, we also provide a step-by-step tutorial on GitHub so other researchers can carry out these analyses using their own data.

## 2 Materials and Experimental Settings

### 2.1 Data

We used two different data sources in our analyses: in sillico and real data. For the in sillico data, different noise structures were chosen to reflect how different configurations of pipelines may interact with the distribution of the data, and the sample size was varied to encompass small to larger scale clinical studies. In the real data analysis, we mimicked how real neuroimaging studies compare an intervention to a reference measurement. Pipelines were selected independently of the intervention data using the healthy/placebo arm ofFrokjaer et al. (2015)using results fromNørgaard, Ganz, et al. (2019), investigating the impact of preprocessing pipelines. The proposed sensitivity analysis was illustrated on the intervention arm of the study where the follow-up value was compared to a reference value taken from a normative serotonergic atlas (Beliveau et al., 2017).

To simulate in sillico data, we considered the simple case of a single brain measurement (R=1), with a single binary exposure (P=1) following a Bernoulli distribution with parameterπ=0.5(i.e., two balanced groups) and no covariates (C=0). LatentYvalues for the brain measurement were simulated using a normal distribution with variance 1 and meanβtimes the exposureXvalues, whereβ=0(null hypothesis) orβ=0.5(alternative hypothesis). The observedYwas simulated for each pipeline, adding pipeline specific noise to the latentY. This noise was simulated using a multivariate normal distribution with mean 0 and variance$\sum_{\text{scenario 1}}$,$\sum_{\text{scenario 2}}$, and$\sum_{\text{scenario 3}}$depending on the scenario (seeSupplementary Material Afor details). In scenario 1, we simulated many pipelines (J=20) with correlated homoscedastic noise; in scenario 2, a few pipelines (J=6) with uncorrelated heteroscedastic noise; and in scenario 3, many pipelines (J=20) with correlated heteroscedastic noise. Scenario 2 and 3 included one pipeline with high signal to noise ratio (SNR), that is, low variance, and many pipelines with low SNR, that is, high variance. Additional simulations with non-normally distributed noise ($\sum_{\text{scenario 4}}$and$\sum_{\text{scenario 5}}$) can be found inSupplementary Material E, investigating the impact of outliers ($\sum_{\text{scenario 4}}$) and skewed data ($\sum_{\text{scenario 5}}$). The sample size was varied fromn=10ton=500in each group, such that the smallest sample size was well below the number of parameters (2×Jmean parameters,$J^{2}$variance-covariance parameters) in scenario 1 and 3 and the asymptotic regime was reached for the largest sample size. We generated 10,000 datasets per scenario and sample size—this provides sufficient precision about the mean, standard deviation, and rejection rate to neglect the Monte Carlo uncertainty. These data will be used to assess the large sample size properties of the procedure (bias, variance, type 1 error control) in finite samples.

To illustrate the use of the proposed sensitivity analysis on real data, we utilize neuroimaging results from a placebo-controlled, double-blinded, clinical study (Frokjaer et al., 2015). The study was registered and approved by the ethics committee for the capital region of Copenhagen (protocol-ID: H-2-2010-108) and registered as a clinical trial:www.clinicaltrials.govunder the trial ID NCT02661789. All subjects provided written informed consent prior to participation, in accordance with The Declaration of Helsinki II. The aim of the study was to assess the association between the emergence of depressive symptoms and change in cerebral serotonin transporter (SERT) availability following a hormonal treatment (P=1). Raw data is available from the CIMBI database (https://nru.dk/index.php?view=category&id=128) upon request under GDPR law, and derived data is available on Github. It consists of structural Magnetic Resonance Imaging (MRI) and Positron Emission Tomography (PET) imaging data for60healthy females who underwent a baseline scan, received either Placebo (n=30) or a GnRHa implant intervention (n=30), and participated in a follow-up scan. SERT availability estimates were modeled and extracted for each subject for 28 subcortical and cortical regions (seeNørgaard, Ganz, et al., 2019for details), and averaged across hemispheres, producing a final sample ofR=14regions per subject and pipeline. These regions (amygdala, thalamus, putamen, caudate, anterior cingulate cortex, hippocampus, orbital frontal cortex, superior frontal cortex, occipital cortex, superior temporal gyrus, insula, inferior temporal gyrus, parietal cortex, and entorhinal cortex) were chosen because they cover the entire brain, and many are target regions in published serotonin transporter (SERT) PET studies. No covariates were considered (C=0).

### 2.2 Multiverse analyses of real data

Five preprocessing steps were used to analyze the data and estimate the SERT availability (outcome measure). These steps include motion correction (with/without), co-registration (4 options), delineation of volumes of interest (3 options), partial volume correction (4 options), and kinetic modeling for quantification of SERT availability (MRTM, SRTM, Non-invasive Logan and MRTM2). More information about the preprocessing choices and software can be found in (Nørgaard, Ganz, et al., 2019). The combination of individual preprocessing steps leads to a number of$J=2\times 3\times 4^{3}=384$possible combinations.

### 2.3 Notation and assumptions

We now introduce notations that will be used to describe the proposed sensitivity analysis (seeSupplementary Material Ffor a summary). We are interested in relatingRbrain measurements ($\text{Y}=(Y_{1},\ldots ,Y_{R})$) toPexposures or treatments ($\text{X}=(X_{1},\ldots ,X_{P})$) accounting forCcovariates ($\text{W}=(W_{1},\ldots ,W_{C})$). We consider a set ofJpipelines used to preprocess the neuroimaging data. For a given pipelinej∈{1,…,J}we fit a statistical model with parameters$\theta_{j}$that relatesYprocessed by pipelinej,X, andW. We then obtain from this model an estimate${\hat{\psi }}_{j}$of the effect of interest (denotedψ). In our real-life example, we use, for each pipeline, a paired t-test to compare the observed change in SERT availability to an atlas value so forj∈{1,…,J},${\hat{\theta }}_{j}=\left({\hat{\psi }}_{j},{\hat{\sigma }}_{j}^{2}\right)$where${\hat{\psi }}_{j}$is the empirical mean and$\sigma_{j}^{2}$the empirical variance of the change in SERT availability (processed with pipelinej) between baseline and follow-up.

We make the following working assumptions: first, the observed data${\left(O_{i}\right)}_{i\in \left\{1,\ldots ,n\right\}}={\left({\text{y}}_{i},{\text{x}}_{i},{\text{w}}_{i}\right)}_{i\in \left\{1,\ldots ,n\right\}}$correspond to independent and identically distributed replicates of(Y,X,W). Second, we have chosen a set of reasonable pipelines, meaning that the estimated effects${\hat{\psi }}_{1},\ldots ,{\hat{\psi }}_{J}$found in the follow-up statistical analysis will converge to the right valueψas the sample size increases. This set can include pipelines distorting the signalY(e.g., adding a fixed value) if that has no consequence, asymptotically, on the estimated effect (the mean change is not biased as the added value cancels out when substracting the baseline value to the follow-up value). Finally, when considering asymptotic results we will consider a fixed numberJof pipelines and let the sample sizenincrease to infinity.

## 3 Proposed Sensitivity Analysis

To be able to draw conclusions across pipelines, we not only need the result of each pipeline but also some information about how they relate. If all pipelines were equally reliable and equally different, we would weight each pipeline equally. If there exists one independent pipeline and a block of correlated pipelines, all equally reliable, then we would weight the independent pipeline more compared with other pipelines. By treating pipelines as black boxes, we can investigate their relation in terms of each observation’s influence on the estimated effects across pipelines,$\hat{\psi }=\left({\hat{\psi }}_{1},\ldots ,{\hat{\psi }}_{J}\right)$. This relation is fully characterized by the joint distribution of the effects. Once estimated, we can extract summaries of this distribution, for example, an average value, and carry out statistical tests, for example testing the compatibility between the observations and the joint distribution that would have been observed under a specific hypothesis.

### 3.1 Estimating the joint distribution across pipelines

The joint distribution could be obtained using a multivariate model, for example, modeling data from all pipelines at once using a mixed model. Because of the complexity of the dependency among pipelines, this is, however, rarely feasible with the available sample size. Instead, and this matches common practice, we perform the same analysis separately for each pipeline and obtain a vector of estimated associations$\hat{\psi }$with their standard errors$\sigma_{\hat{\psi }}=\left(\sigma_{{\hat{\psi }}_{1}},\ldots ,\sigma_{{\hat{\psi }}_{J}}\right)$. Using tools from the semi-parametric theory (seeKennedy, 2017andTsiatis, 2006for more details), we can approximate the influence of each observation on the estimate by a random variable called the influence function, denoted$\varphi_{{\hat{\psi }}_{j}}$for pipelinej, and satisfying:

$$\sqrt{n}\left({\hat{\psi }}_{j}-\psi \right)=\frac{1}{\sqrt{n}}\sum_{i=1}^{n}\varphi_{\psi_{j}}\left(O_{i}\right)+o_{p}(1)$$

where$o_{p}(1)$denotes a residual term that convergences toward zero in probability as the sample size tends to infinity. As shown inSupplementary Material B,$\varphi_{{\hat{\psi }}_{j}}$has a simple expression when${\hat{\psi }}_{j}$is the empirical mean or an element of a maximum likelihood (ML) estimator. Since this decomposition applies to all pipelines, we get from the multivariate central limit theorem that the joint distribution of the estimates is asymptotically multivariate normal. It has meanψand its variance-covariance, denoted$\sum_{\hat{\psi }}$, is the same as the one of${\hat{\varphi }}_{\hat{\psi }}=\left(\varphi_{{\hat{\psi }}_{1}},\ldots ,\varphi_{{\hat{\psi }}_{J}}\right)$divided byn. Note that with limited number of observations, typically whenn<J, the estimated variance-covariance${\hat{\Sigma }}_{\hat{\Psi }}$based on the estimated influence function is not guaranteed to be positive definite.

### 3.2 Testing hypotheses across pipelines

The global null hypothesis “no effect across all pipelines” can be tested using a max-test approach: more extreme realizations would correspond to larger values of the maximum statistic${\hat{\text{t}}}_{\text{max}}=\text{max}\left(\left|{\hat{t}}_{1}\right|,..,\left|{\hat{t}}_{J}\right|\right)$where|.|denotes the absolute value and${\hat{t}}_{j}=\frac{{\hat{\psi }}_{j}}{\sigma_{{\hat{\psi }}_{j}}}$. A p-value may, therefore, be computed by integrating the joint density under the null hypothesis outside of the domain$D\left({\hat{\text{t}}}_{\text{max}}\right)={\left[-{\hat{\text{t}}}_{max},\;\;{\hat{\text{t}}}_{max}\right]}^{⊗J}$(seeSupplementary Fig. BinSupplementary Material C). Here, we use the notation${\left[a,b\right]}^{⊗J}=\prod_{j=1}^{J}\left[a,b\right]$that represents the Cartesian product betweenJintervals[a,b]. The value$t_{c}$, such that the integral outside$D\left(t_{c}\right)$equalsα, provides a critical threshold for the estimated test statistics$\left(\left|{\hat{t}}_{1}\right|,..,\left|{\hat{t}}_{J}\right|\right)$. This threshold can also be used to derive confidence intervals.

The null hypothesis “at least one pipeline with no effect” is an intersection union test. As such, it can be rejected if and only if all the un-adjusted p-values relative to each pipeline are belowαor equivalently if the largest un-adjusted p-value is belowα.

The proportion of pipelines where there is evidence for an effectηcan be estimated as${\hat{\eta }}_{𝟙}=\frac{1}{J}\sum_{j=1}^{J}{𝟙}_{{|\hat{t}}_{j}|<t_{c}}$where${\%}_{.}$denotes the indicator function. One drawback with this non-parametric estimator is that it is a non-smooth function of${\hat{t}}_{j}$, making the associated uncertainty difficult to evaluate. Instead, one can use a parametric approach, assuming normally distributed test statistics:

$$\begin{array}{ccc} {\hat{\eta }}_{\Phi }\;=\frac{1}{J}\;\sum_{j=1}^{J}1\;-\;\mathbb{P}[-t_{c}\;<{\hat{t}}_{j}\;<t_{c}]=1-\;\frac{1}{J}\sum_{j=1}^{J}\Phi \left(t_{c}\;-{\hat{t}}_{j}\right)-\Phi \left(-t_{c}\;-{\hat{t}}_{j}\right) & & \end{array}$$

which is a smooth (but complex) function of the model parameters. Here,Φrefers to the cumulative distribution function of a standard normal distribution. The uncertainty about the estimator can, therefore, be derived using a non-parametric bootstrap or a delta method where$Var\left({\hat{\eta }}_{\Phi }\right)=\frac{\partial {\hat{\eta }}_{\Phi }}{\partial \Theta }\sum_{\hat{\Theta }}\frac{\partial {\hat{\eta }}_{\Phi }^{\;⊺}}{\partial \Theta }$, and$\Theta ={\left(\theta_{j}\right)}_{j\in \left\{1,\ldots ,J\right\}}$is the set of parameters of the statistical model across pipelines.

### 3.3 Visualizing the heterogeneity across pipelines

In section 3.1 , it was stated that the estimated associations are, asymptotically, normally distributed. They can, therefore, be summarized by their expectation and variance-covariance matrix (i.e., standard errors and correlation matrix). In this work, we suggest two graphical displays to visualize the heterogeneity of the results across pipelines:

- A heatmap of the estimated correlation among estimates, obtained by converting${\hat{\Sigma }}_{\hat{\Psi }}$into a correlation matrix.^1^
- A forest plot displaying$\left({\hat{\psi }}_{1},\ldots ,{\hat{\psi }}_{J}\right)$and$\left({\hat{\sigma }}_{{\hat{\psi }}_{1}},\ldots ,{\hat{\sigma }}_{{\hat{\psi }}_{J}}\right)$through the confidence intervals, possibly using the previously established re-ordering.

### 3.4 Estimating a global effect across pipelines

Several methods can be used for estimating a global effect across pipelines. A naive method would be to compute the mean of the estimated associations:

$$\begin{array}{ccc} {\hat{\Psi }}_{\text{average}}=\frac{1}{J}\sum_{j=1}^{J}{\hat{\psi }}_{j} & & \end{array}$$

This estimator will, however, not be efficient if some pipelines lead to more precise estimates, that is, the elements in$\left(\sigma_{{\hat{\psi }}_{1}},\ldots ,\sigma_{{\hat{\psi }}_{J}}\right)$are not equal. Intuitively, we would like to pool the estimates with weights inversely proportional to the standard errors such that we put more weight on precise estimates:

$$\begin{array}{ccc} {\hat{\Psi }}_{\text{pool-se}}=\sum_{j=1}^{J}w_{j}^{se}{\hat{\psi }}_{j}\text{ where }w_{j}^{se}=\frac{1/\sigma_{{\hat{\psi }}_{j}}^{2}}{\sum_{j=1}^{J}1/\sigma_{{\hat{\psi }}_{j}}^{2}} & & \end{array}$$

However, we also need to take into account the correlation between estimates. Indeed, perfectly correlated estimates should weight as if there was only one estimate. To do so, we can use the following GLS estimator of the global effect:

$$\hat{\Psi }\text{GLS}={\left(1^{T}{\hat{\Sigma }}_{\hat{\Psi }}^{-1}1\right)}^{-1}1^{T}{\hat{\Sigma }}_{\hat{\Psi }}^{-1}\hat{\Psi }=\sum_{j=1}^{J}W_{j}^{GLS}{\hat{\Psi }}_{J}$$ (1)

where1is a column vector filled with ones and$\sum_{j=1}^{J}w_{j}^{\text{GLS}}=1$. Indeed,equation 1can be shown to be equivalent to performing a spectral decomposition of${\hat{\Sigma }}_{\hat{\Psi }}$, and using the eigenvectors to combine estimates into independent components that can be pooled according to weights proportional to the eigenvalues (Supplementary Material D1). This is used when$\sum_{\hat{\psi }}$is singular to compute${\hat{\Psi }}_{\text{GLS}}$, by restricting the spectral decomposition to the eigenvalues above a given threshold ($\in =10^{-10}$). In the simple case whereR=1,P=1,C=0, brain measurements are jointly normally distributed,Xis binary, and there are no missing data, the GLS estimator can be shown to be asymptotically efficient (Supplementary Material D2).

Cox et al. (2006)studied a similar estimator whenJ=2and found that, under unequal variance, the global estimate can be outside of the range of the (pipeline specific) estimates. As a remedy, we propose a constrained GLS estimator, denoted${\hat{\Psi }}_{\text{constrained GLS}}$, which, in addition to have weights that sum up to 1, constrains the weight of each estimate to be at most 1 in absolute value:

$$\begin{array}{ccc} w_{j}^{\text{constrained GLS}}=\{\begin{array}{cc} w_{j}^{\text{GLS}} & {\text{if max}}_{j\in \left\{1,\;\ldots ,J\right\}}\left|w_{j}^{GLS}\right|\leq 1\\ \frac{w_{j}^{GLS}}{\kappa +{\text{max}}_{j\in \left\{1,\;\ldots ,J\right\}}\;\left|w_{j}^{GLS}\right|}+\frac{1}{J}\left(1-\frac{1}{\kappa +{\text{max}}_{j\in \left\{1,\;\ldots ,J\right\}}\;\left|w_{j}^{GLS}\right|}\right) & \text{otherwise} \end{array} & & \end{array}$$

whereκis chosen to satisfy the constraint (Supplementary Material D3). These constrained weights can be seen as a regularization of the GLS weights (first term) toward the weights for the mean (second term) with regularization coefficient$\frac{1}{\kappa +{\text{max}}_{j\in \left\{1,\;\ldots ,J\right\}}\;\left|w_{j}^{GLS}\right|}$$=\frac{J-1}{J{\text{ max}}_{j\in \left\{1,\;\ldots ,J\right\}}\;\left|w_{j}^{GLS}\right|-1}$. This regularization coefficient is 1 when the largest weight is 1 (${\hat{\Psi }}_{\text{constrained GLS}}={\hat{\Psi }}_{\text{GLS}}$) and decreases toward 0 as the largest weight increases toward infinity (${\hat{\Psi }}_{\text{constrained GLS}}\to {\hat{\Psi }}_{\text{average}}$).

Note that if some of the pipelines may induce some bias, the previous approaches will propagate this bias and therefore be unsatisfactory. Systematic differences between pipelines can be investigated by comparing the estimates between pipelines, for example,${\hat{\psi }}_{j}-{\hat{\psi }}_{j^{\prime }}$and using${\hat{\Sigma }}_{\hat{\Psi }}$to obtain the corresponding uncertainty:$Var\left[{\hat{\psi }}_{j}-{\hat{\psi }}_{j^{\prime }}]=Var[{\hat{\psi }}_{j}]+Var[{\hat{\psi }}_{j^{\prime }}\right]-2\mathbb{C}ov\left({\hat{\psi }}_{j},{\hat{\psi }}_{j^{\prime }}\right)$.

## 4 Results

### 4.1 Simulated multiverse analysis for normally distributed data

We compare the performance of the proposed estimators when using unbiased pipelines on simulated data. For each dataset$\sum_{\text{scenario 1}}$,$\sum_{\text{scenario 2}}$, and$\sum_{\text{scenario 3}}$, we computed the four previously described estimators of the global effect,${\hat{\Psi }}_{\text{average}}$,${\hat{\Psi }}_{\text{pool-se}}$,${\hat{\Psi }}_{\text{GLS}}$, and${\hat{\Psi }}_{\text{constrained GLS}}$. P-values for${\hat{\Psi }}_{\text{pool-se}}$,${\hat{\Psi }}_{\text{GLS}}$, and${\hat{\Psi }}_{\text{constrained GLS}}$were computed, neglecting the uncertainty of the weights ($w_{j}^{\text{se}}$,$w_{j}^{\text{GLS}}$,$w_{j}^{\text{constrained GLS}}$).

#### 4.1.1 Weights (global effect)

**Fig. 1.**
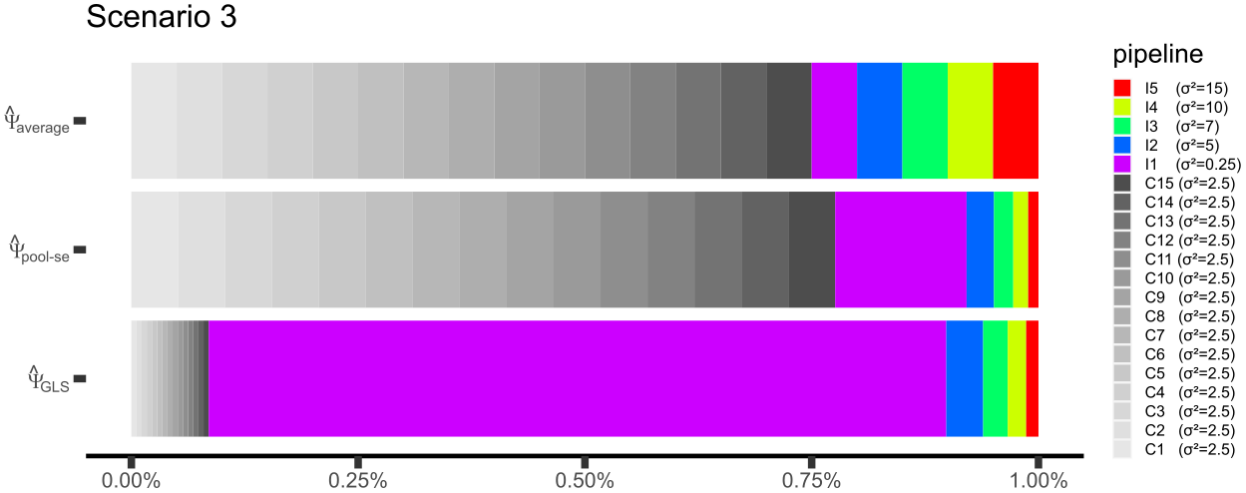
Large sample weights used by${\hat{\Psi }}_{\text{average}}$,${\hat{\Psi }}_{\text{pool-se}}$, and the GLS estimators (${\hat{\Psi }}_{\text{GLS}}$or${\hat{\Psi }}_{\text{constrained GLS}}$) to combine the pipeline-specific estimates in scenario 3. Weights relative to the correlated pipeline are shown in shades of gray (first 15 blocks); weights relative to the independent pipeline are shown using rainbow colors (last 5 blocks). In the legend, the variance for a given pipeline effect is indicated in parentheses.

Based on$\sum_{\text{scenario 1}}$,$\sum_{\text{scenario 2}}$, and$\sum_{\text{scenario 3}}$, we can compute how${\hat{\Psi }}_{\text{average}}$,${\hat{\Psi }}_{\text{pool-se}}$, and${\hat{\Psi }}_{\text{GLS}}$(or${\hat{\Psi }}_{\text{constrained GLS}}$) would weight the results from each pipeline if the variance-covariance matrix of the pipeline estimates was known (Fig. 1). In scenario 1,${\hat{\Psi }}_{\text{average}}$and${\hat{\Psi }}_{\text{pool-se}}$would provide equal weight to all pipelines with a weight of 5%, while${\hat{\Psi }}_{\text{GLS}}$would weight by 1.88% the correlated pipelines and by 14.37% the uncorrelated pipelines (in this paragraph, all weights are rounded for readability). In scenario 2,${\hat{\Psi }}_{\text{average}}$equally weights all pipelines by 16.67% while${\hat{\Psi }}_{\text{pool-se}}$and${\hat{\Psi }}_{\text{GLS}}$would use the following weights: 18.75%, 52.51%, 10.94%, 7.72%, 5.97%, and 4.10%, favoring the high SNR pipeline. In scenario 3,${\hat{\Psi }}_{\text{average}}$would equally weight all pipelines by 5%,${\hat{\Psi }}_{\text{pool-se}}$would weight each correlated pipeline by 5.17% and the remaining pipelines by 14.48% (high SNR pipeline), 3.02%, 2.13%, 1.65%, and 1.13%, while${\hat{\Psi }}_{\text{GLS}}$would weight by 1.65% the correlated pipelines and by 48.61% (high SNR pipeline), 10.13%, 7.15%, 5.52%, and 3.80% the remaining pipelines.

#### 4.1.2 Point estimate (global effect)

The average estimate of the global effect estimators (${\hat{\Psi }}_{\text{average}}$,${\hat{\Psi }}_{\text{pool-se}}$,${\hat{\Psi }}_{\text{GLS}}$,${\hat{\Psi }}_{\text{constrained GLS}}$) was close to the true value for all sample sizes and all scenarios, typically±0.005, and the largest discrepancy was 0.015 (${\hat{\Psi }}_{\text{GLS}}$, n=10 per group, scenario 3, alternative hypothesis).

#### 4.1.3 Point estimate (proportion)

Under the null, the estimated proportion of pipelines with evidence for an effect ranged between 5.35% and 6.79% based on${\hat{\eta }}_{\Phi }$and between 0.50% and 1.71% based on${\hat{\eta }}_{\%}$.${\hat{\eta }}_{\Phi }$showed no obvious trend with sample size, whereas${\hat{\eta }}_{\%}$showed a small decrease with the sample size (upper panel ofSupplementary Fig. C). Under the alternative, the proportion of pipelines dramatically increased with the sample size for both estimators (upper panel ofFig. 3): typically from 10% or below whenn=10per group to 99% in scenario 1, 65% in scenario 2, and around 80% in scenario 3 withn=500per group. The “usual” estimator${\hat{\eta }}_{\%}$exhibited a smaller value at low sample size but a steeper increase compared to the proposed estimator${\hat{\eta }}_{\Phi }$.

#### 4.1.4 Variance (global effect)

Similar results were obtained forβ=0andβ=0.5, so we will only discuss the former case (upper panels ofFig. 2). In scenario 1,${\hat{\Psi }}_{\text{average}}$and${\hat{\Psi }}_{\text{pool-se}}$showed similar variability, whereas the variability of${\hat{\Psi }}_{\text{GLS}}$was higher in very small samples (standard deviation: +156% forn=10, +11% forn=25) and lower for larger sample sizes (e.g., -11% forn=500). In scenario 2,${\hat{\Psi }}_{\text{pool-se}}$and${\hat{\Psi }}_{\text{GLS}}$showed similar variability (slightly higher for${\hat{\Psi }}_{\text{GLS}}$in low sample sizes and slightly lower afterward), both smaller than the variability of${\hat{\Psi }}_{\text{average}}$, for example, -9.93% forn=10and -24.86% forn=500for${\hat{\Psi }}_{\text{GLS}}$. Results in scenario 3 were similar to scenario 1 up to a larger decrease in variability in large samples for${\hat{\Psi }}_{\text{GLS}}$(-27.75% for n=500). The variability of${\hat{\Psi }}_{\text{constrained GLS}}$was similar to one of${\hat{\Psi }}_{\text{GLS}}$except in small samples where it was smaller and closer to the one of${\hat{\Psi }}_{\text{average}}$.

**Fig. 2.**
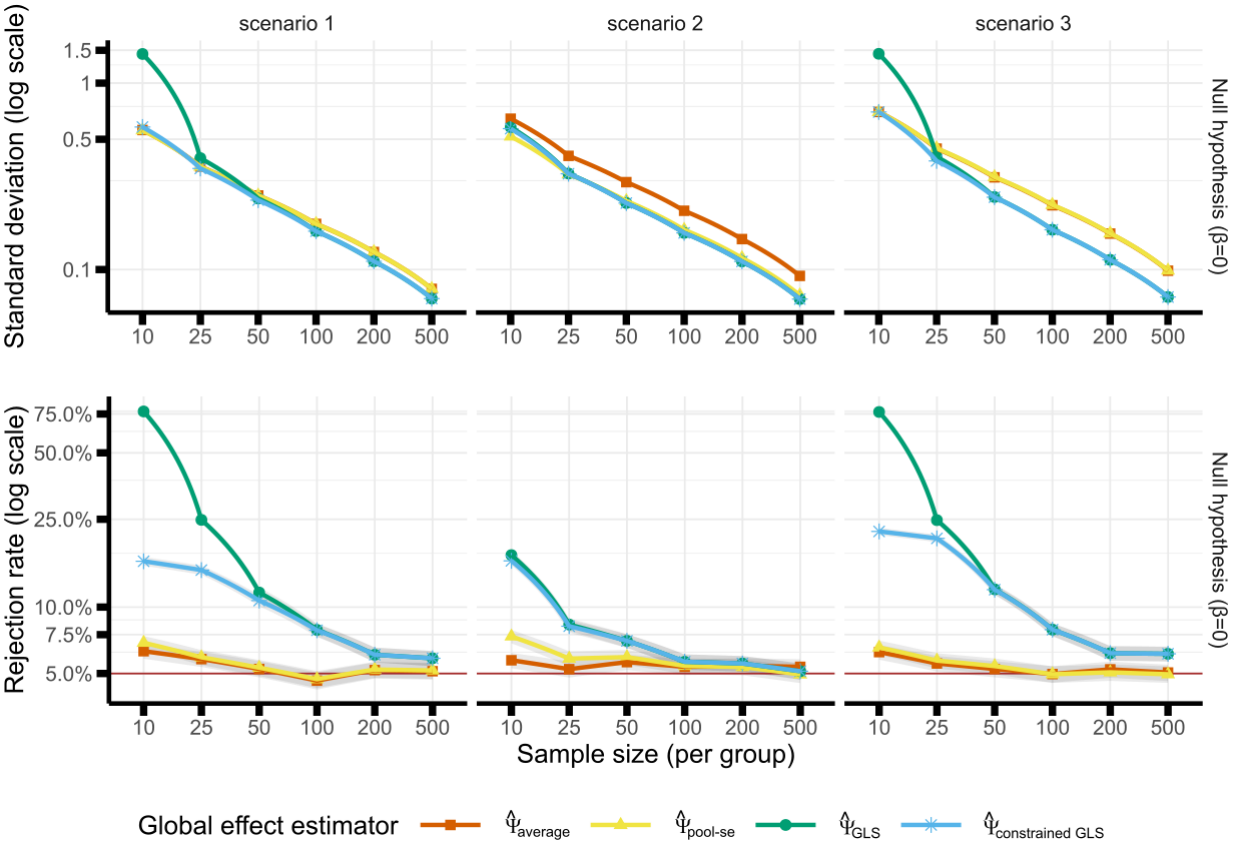
Upper panel: empirical standard deviation of the estimated common effect for each estimator, scenario, and sample size under the null hypothesis. For large samples, the green line (GLS) is covered by the blue line (constrained GLS) in all scenarios. In scenarios 1 and 3, the red line (average) is covered by the yellow line (pool-se). In scenario 1, the GLS estimator has the highest standard deviation in small samples and the GLS and GLS constrained have the lowest standard deviation (approx. 12% lower compared to the average). Lower panel: rejection rate under the null hypothesis (i.e., type 1 error). Shaded area represents the Monte Carlo uncertainty.

#### 4.1.5 Variance (proportion)

The proposed estimator${\hat{\eta }}_{\Phi }$had a lower variability compared to the usual estimator${\hat{\eta }}_{1}$in all scenarios and across sample sizes under the alternative hypothesis (except in scenario 1,n=500): the reduction in standard deviation ranged from 5.67% to 36.94% (lower panels ofFig. 3).

**Fig. 3.**
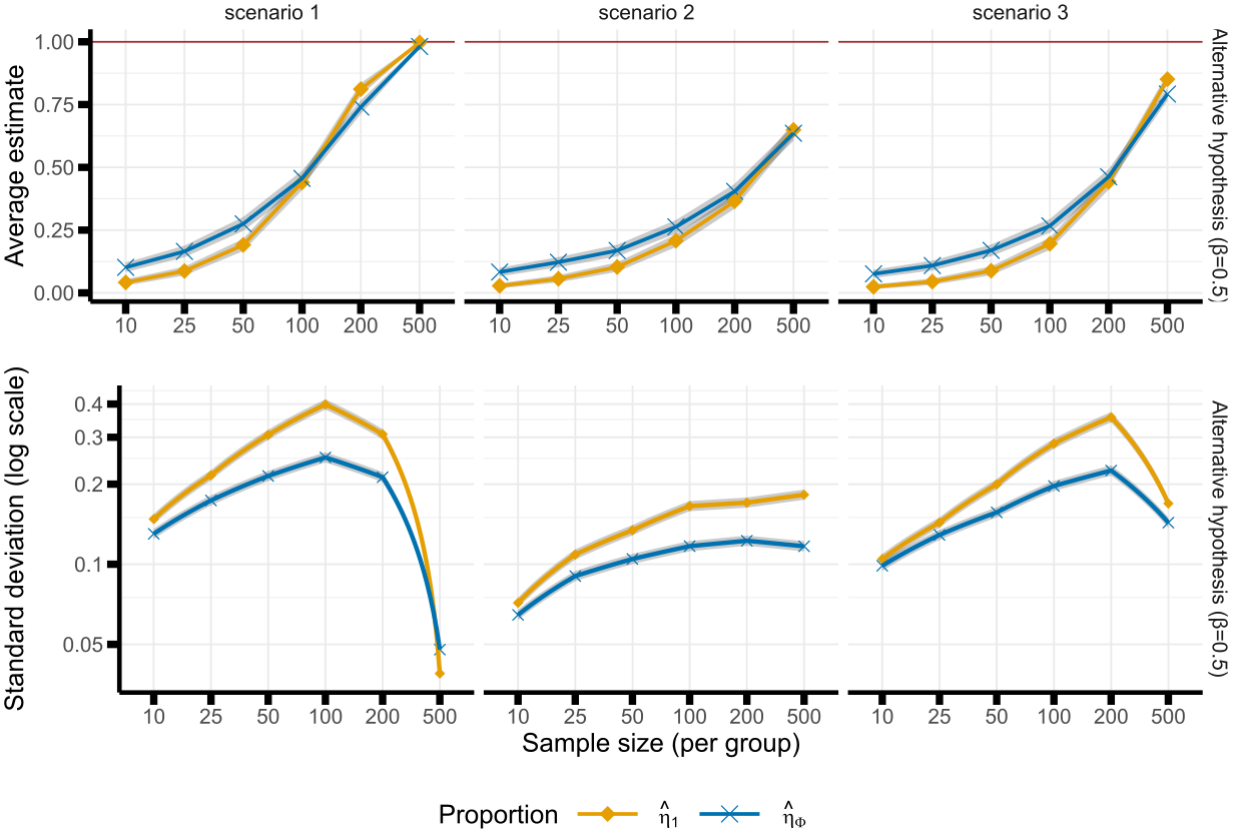
Empirical mean (upper panel) and standard deviation (lower panel) of the two proportion estimators (${\hat{\eta }}_{\%}$and${\hat{\eta }}_{\Phi }$) for each scenario, and sample size under the alternative hypothesis. Shaded area represents the Monte Carlo uncertainty.

#### 4.1.6 Type 1 error (global effect)

Across all scenarios, the type 1 errors for${\hat{\Psi }}_{\text{average}}$and${\hat{\Psi }}_{\text{pool-se}}$were well controlled except in very small samples where small deviations from the nominal level were observed (maximum of 6.31% for${\hat{\Psi }}_{\text{average}}$and 7.36% for${\hat{\Psi }}_{\text{pool-se}}$, lower panels ofFig. 2). When neglecting the uncertainty about the weights, the type 1 error control of${\hat{\Psi }}_{\text{GLS}}$and${\hat{\Psi }}_{\text{constrained GLS}}$was only controlled for large samples, that is, forn=500; large type 1 error rates were found in very small samples (e.g., 22.02% for${\hat{\Psi }}_{\text{constrained GLS}}$withn=10).

### 4.2 Simulated multiverse analysis for non-normal distributions

**Fig. 4.**
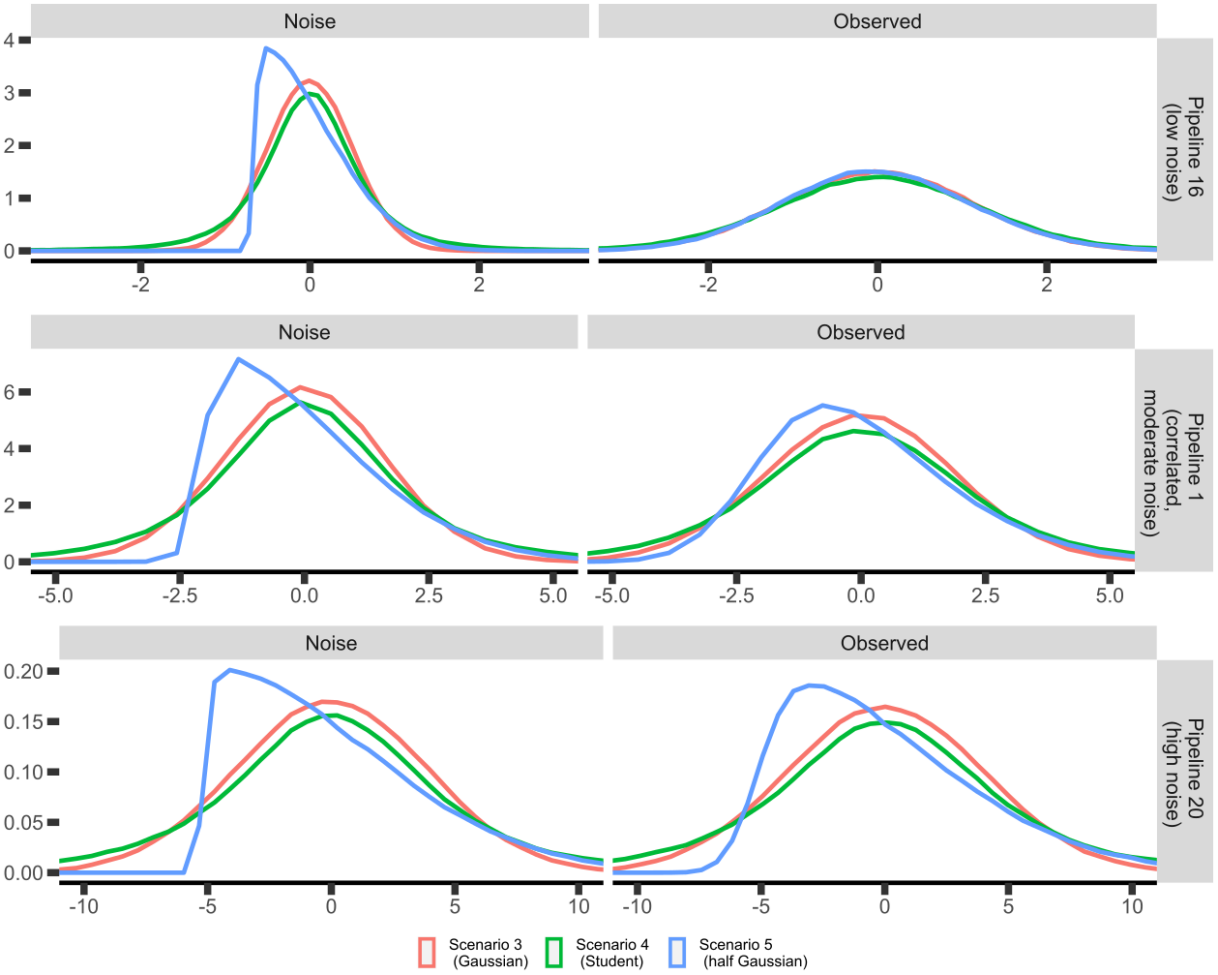
Density of the (marginal) distribution of the noise (left panels) and of the observed values (right panels) for three pipelines. Colors refer to different scenarios, that is, different noise distributions.

While the previous section evaluated the performance of the sensitivity analysis assuming ground-truth data generated from a multivariate normal distribution, this assumption is often violated in real neuroimaging data (especially with patient data). To address this issue, we expanded the simulation scenarios to include non-normal data (scenario 4 and 5), allowing us to understand how well the sensitivity analysis works, how these data distributions interact with sample size, and what additional constraints are necessary to govern the usage of the sensitivity analyses. More specifically, we created two additional simulations largely corresponding to scenario 3 (many pipelines with correlated heteroscedastic noise), but with different noise distributions. Scenario 4 used a noise distribution following a Student’s t-distribution with 5 degrees of freedom, resulting in a distribution with heavy tails to reflect the inclusion of outliers. Scenario 5 used a noise distribution following a half-normal distribution, resulting in a heavily skewed non-negative distribution, with most of the density centered around 0.Figure 4highlights the differences between scenario 3, 4, and 5, across different noise levels. Additional simulation details for scenario 4 and 5 can be found in theSupplementary Material E.

Notably, the results for these additional scenarios were similar to the multivariate normal cases in terms of bias, variability, and rejection rate (Supplementary Fig. E). However, while these additional scenarios may not fully represent real-world data, they show the robustness of the proposed sensitivity analysis to assumptions about the residual distribution. Intuitively, they benefit from the law of large numbers, that is, for large enough sample sizes they will behave as expected. Worse low sample size performances with non-Gaussian noise distributions is expected, which is in line with some of the simulation results (e.g., bias in scenario 5,Supplementary Fig. D). The difference between the Gaussian and non-Gaussian case is not striking though, and factors such as sample size seem to be driving the behavior of the estimators.

### 4.3 Application to real-world multiverse analysis

We illustrate the statistical sensitivity analysis with real data described inSection 2.1in which the SERT availability was assessed after a drug intervention and compared to normative values. We start by studying the behavior of the four statistical estimators (pooled GLS, pooled constrained GLS, pooled average and pooled SE) to estimate a common effect across pipelines for the given null hypothesis for a single subcortical region - amygdala - first, reported as a forest plot inFigure 5(left panel). The dashed vertical line inFigure 5represents the normative value, and all horizontal error bars represent the estimated effect (mean and 95% CI) for a given pipeline and estimator. Across a reasonable set of preprocessing pipelines, three of the eight selected reject the null hypothesis (as indicated by the non-overlapping CI with the normative value) with estimated percent differences between groups ranging between -9% (pipeline 6) and 2.5% (pipeline 1). The pooled constrained GLS, pooled SE and pooled average all fail to reject a common effect across pipelines, whereas the GLS estimator rejects the null hypothesis. However, when inspecting the pipeline weights for the GLS estimator for these data, it assigned a very high weight to four pipelines (i.e., weight above 1 in absolute values), leading to an unreliable estimate as illustrated by the large standard deviation found in the simulation study for low sample sizes (Fig. 2). The constrained GLS estimator did not exhibit this problem and had weights between -0.79 and 1. We also visualize the effect of the four global effect estimators across the nine cortical regions. This is shown inFigure 6. Here, we can observe a similar pattern, where the pooled GLS estimator exhibited the largest deviations from the expected value based on the SERT normative values, whereas the pooled constrained GLS estimator is close to the pooled SE and pooled average.

**Fig. 5.**
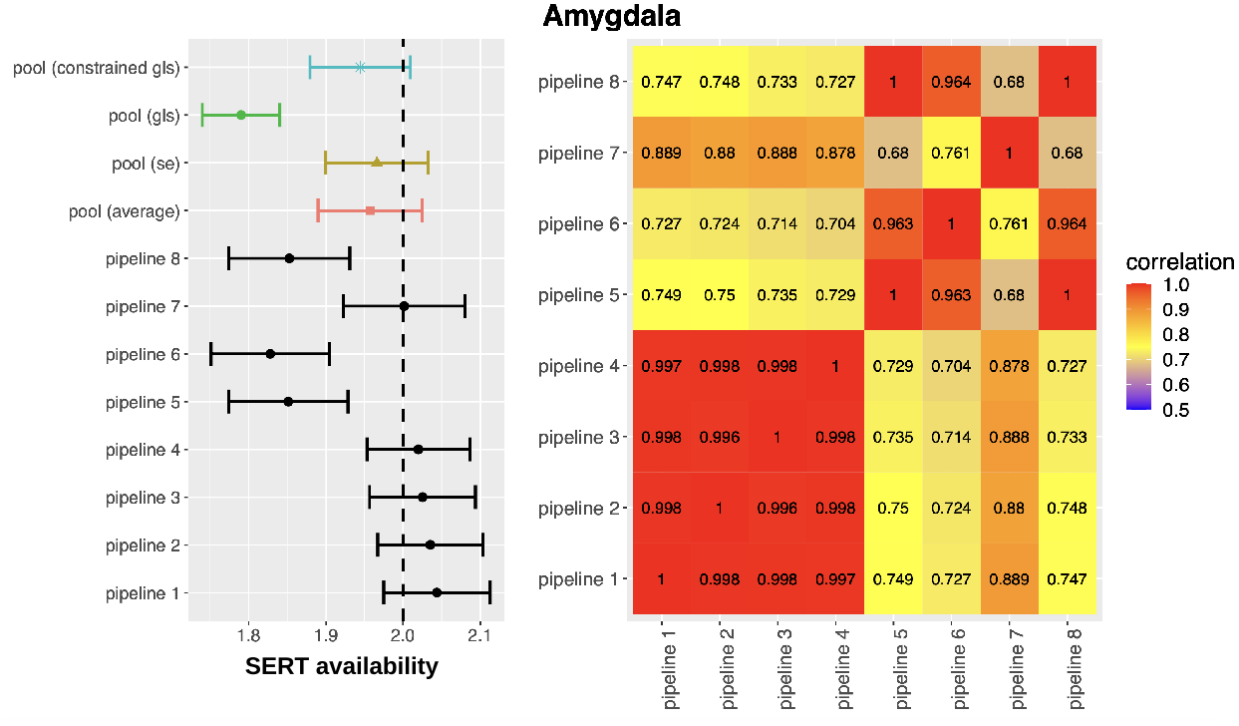
Left panel: forest plot of the estimated SERT availability in the amygdala for the intervention group (point and full line) versus the normative values (dashed line) for each pipeline and the four proposed pooled estimators. Right panel: correlation of the estimated SERT availability between pipelines. Pipeline 1: with motion correction (MC), boundary based registration (BBR) using the time-weighted average PET image (twa), and MRTM2 as kinetic modeling choice to estimate SERT availability. Pipeline 2: MC, normalized mutual information registration (NMI) using twa, MRTM2. Pipeline 3: MC, BBR using the average PET image, and MRTM2. Pipeline 4: MC, NMI using the average PET image, and MRTM2. Pipeline 5: MC, BBR_twa, and MRTM. Pipeline 6: no motion correction (nMC), BBR_twa, and MRTM. Pipeline 7: nMC, BBR_twa, and MRTM2. Pipeline 8: MC, BBR_twa, and SRTM.

**Fig. 6.**
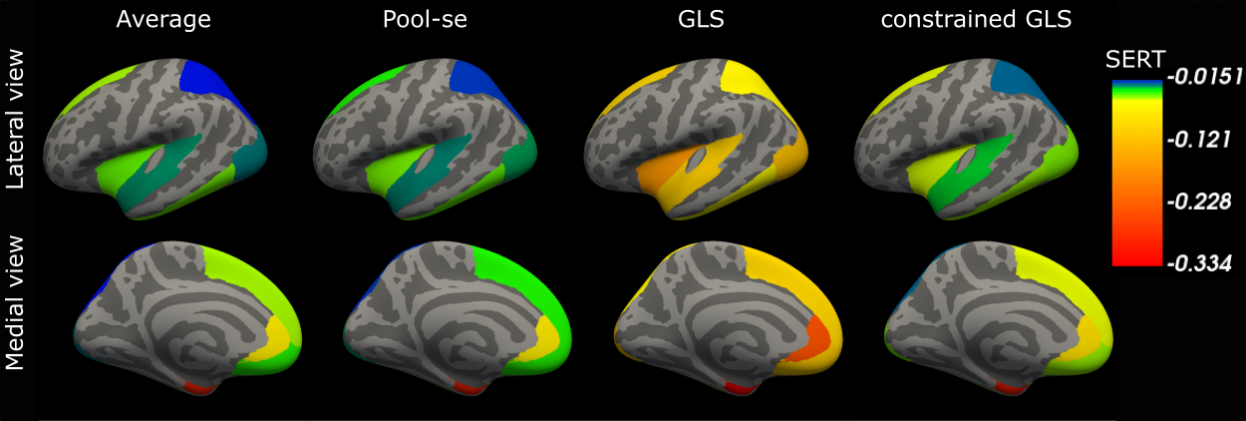
Estimated difference (intervention - normative values) of SERT availability across pipelines for the four global effect estimators on the common inflated FreeSurfer surface (left hemisphere; lateral view, upper and medial view, lower). Note, only the nine cortical regions of interest are visualized.

Figure 5(right panel) shows a heatmap for the estimated correlations across preprocessing pipelines, ranging from 0.68 to 1. Pipelines 1-4 show a very high correlation with each other (only varying the registration choices), whereas pipelines 5, 6, and 8 are equally correlated with each other (different kinetic models), and pipeline 7 is somewhat in between (no motion correction). The heatmap captures important differences in the correlation structure between pipelines, suggesting that not all pipelines perform similarly with moderate levels of unexplained variance. Pipelines 4 to 8 exhibit numerically smaller correlation compared to pipelines 1 to 4 (and similar variance), which explains why GLS estimators produce estimates with lower numerical values compared to pooled estimators ignoring the correlation, as GLS estimators assign more weight to the last four pipelines.

Notably, no correction for multiple comparisons was carried out across regions and pipelines at this point. The rationale for not including this (as should otherwise always be carried out) is that we wanted to make our analysis as comparable as possible to the Neuroimaging Analysis Replication and Prediction Study (Botvinik-Nezer et al., 2020), where each participating institution analyzed the data using their own established pipeline and tested only a single region in a hypothesis-driven fashion. Future work should address how adequate correction for multiple comparisons should be carried out across multiple estimators and pipelines.

## 5 Discussion

The proposed statistical framework leverages information about the joint distribution of the test statistics across pipelines to provide insights about the impact of preprocessing strategies. In particular, we introduce a new estimator of the proportion of pipelines with evidence for an effect (${\hat{\eta }}_{\Phi }$) and a new estimator of the effects across pipelines (${\hat{\Psi }}_{\text{constrained GLS}}$), able to pool correlated and heteroschedastics estimates while exhibiting reasonable small sample size properties. This information can complement a standard forest plot, as exemplified inFigure 5where we added a heatmap of the correlation between pipelines.

The simulation results show that while${\hat{\Psi }}_{\text{GLS}}$outperforms standard pooling techniques in large samples (${\hat{\Psi }}_{\text{average}}$or${\hat{\Psi }}_{\text{pool-se}}$), it is not suited for the commonly observed cases in neuroimaging studies of smaller N (number of subjects) relative to the large P (number of pipelines). The proposed alternative estimator (${\hat{\Psi }}_{\text{constrained GLS}}$) can be seen as regularizing the GLS estimator toward the empirical average in small samples. Even though this considerably improves the small sample performance of the estimator, other regularization approaches may lead to further gain. An alternative approach could be to regularize the estimated variance-covariance matrix between pipeline-specific estimates, for example, using graphical lasso. However, this is challenging since the correlation structure among pipelines is typically complex and non-sparse.

The proposed estimator of the proportion of pipelines (${\hat{\eta }}_{\Phi }$) showed lower variability in the simulation studies which should benefit statistical power. It is also intuitively appealing due to less sensitivity to the threshold chosen for statistical significance compared to averaging the number of pipelines for which the p-value was below, say, 0.05.

In the real-world application, we observed that all estimators could be readily applied, and three of them performed as expected based on the results from individual pipelines. Only the${\hat{\Psi }}_{\text{GLS}}$estimator performed differently and was the only one rejecting the null hypothesis hinting at an effect across pipelines. This is, though, due to the estimator not being able to fit the weights properly in small sample sizes. Since neuroimaging studies in general and especially in PET are rarely beyond sample sizes ofn>50(Szucs & Ioannidis, 2020), other estimators should be used. We recommend using${\hat{\Psi }}_{\text{pool-se}}$or${\hat{\Psi }}_{\text{constrained GLS}}$: the latter when pipelines are not similarly related and the sample size is moderate to large, otherwise the former.

The framework that we are proposing is not without limitations. Fast and accurate estimation of statistical uncertainty is still being investigated. One attempt, neglecting the uncertainty of the weights, is numerically fast but unreliable in small samples or with a large number of pipelines for${\hat{\Psi }}_{\text{GLS}}$and${\hat{\Psi }}_{\text{constrained GLS}}$. For the moment, resampling methods such as non-parametric bootstrap or permutation test, while more computationally demanding, are recommended.

The underlying assumption of combining results across pipelines in our analysis is that all pipelines are unbiased and positively correlated (i.e., they all estimate the “same” effect). This can, however, only sometimes be guaranteed. For example, in the well-known NARPS analysis (Botvinik-Nezer et al., 2020), the effect detected by different groups was, for a few cases, in the opposite direction (which is not typical of multiverse analyses). Alternative approaches, for example, STAPLE (Dafflon et al., 2022), could be used to reduce the bias of the pooled estimators by assuming a majority of unbiased pipelines or identifying clusters of pipelines and pooling pipeline-specific estimates within clusters. Finally, our proposed framework currently only considers a single region at a time and not multiple regions of interest jointly. Previous work has shown that optimal preprocessing strategies for single regions exist (Nørgaard, Ganz, et al., 2019), but also that multiple regions of interest are correlated within-subject (Eklund et al., 2016;Ganz et al., 2020). Therefore, future work is still needed to further jointly investigate and summarize the complex and nested dependence structure across multiple regions, and how it interacts with multiple preprocessing strategies.

## 6 Conclusion

In this work, we have developed a statistical sensitivity analysis that can quantify the impact of different preprocessing choices on subsequent statistical analyses. As has been reported in previous studies, we observe that the influence of preprocessing pipelines on subsequent statistical analysis can be quite large. Hence, we provide tools for statistical analyses that can aggregate multiple analyses of the same data. We introduced a new pooling estimator across pipelines (${\hat{\Psi }}_{\text{constrained GLS}}$) but also discuss how to estimate the proportion of pipelines with evidence for an effect and test hypotheses across pipelines, such as “no effect across all pipelines” or “at least one pipeline with no effect”. Code to reproduce all simulations and figures, including a step-by-step tutorial, is available athttps://github.com/openneuropet/multiverse_tools/tree/main.

## Supplementary Material

Supplementary Material

## Data Availability

The script to re-create the in silico data is available on GitHub (https://github.com/openneuropet/multiverse_tools/tree/main/sensitivity_analysis-simulation). The data for the real-world example originate from a clinical study byFrokjaer et al. (2015). The study was registered and approved by the ethics committee for the capital region of Copenhagen (protocol-ID: H-2-2010-108) and registered as a clinical trial:www.clinicaltrials.govunder the trial ID NCT02661789. All subjects provided written informed consent prior to participation, in accordance with The Declaration of Helsinki II. Data are available from the CIMBI database (https://nru.dk/index.php?view=category&id=128) upon request. Code implementing the proposed methods is available on CRAN in the LMMstar package (https://cran.r-project.org/web/packages/LMMstar/index.html). Code for the simulation study and the application is available on GitHub (https://github.com/openneuropet/multiverse_tools/tree/main/sensitivity_analysis-simulation).

## Footnotes

1 Re-ordering the pipelines may be useful to better visualize blocks of pipelines that are particularly correlated. This can, for instance, be performed by converting the correlation matrix to a dissimilarity matrix and then using hierarchical clustering.
