# Supplementary material for: A sensitivity analysis of preprocessing pipelines: Toward a solution for multiverse analyses
Source: Imaging Neurosci (Camb). 2025 Apr 28;3:imag_a_00523. doi: 10.1162/imag_a_00523 (PMC12319823; doi:10.1162/imag_a_00523)
Supplement: Supplementary Material [file imag_a_00523-supp.pdf]

Supplementary material for the article "A sensitivity analysis of preprocessing pipelines: toward a solution for multiverse analyses"

Brice Ozenne, Martin Nørgaard, Cyril Pernet, Melanie Ganz

A: Parameterization of each scenario in the simulation study

Scenario 1: 20 pipelines were considered, all with variance 1. 15 of the 20 pipelines were equally highly correlated (correlation of 0.95) and independent of the remaining 5 pipelines, themself independent of each other (correlation of 0). The corresponding covariance matrix is displayed below:

$$\Sigma_{\text{scenario 1}} = \begin{bmatrix} R_15 & O \\ O & I_6 \end{bmatrix} = \begin{bmatrix} 1.00 & 0.95 & \dots & 0.95 & 0.95 & 0 & \dots & \dots & \dots & 0 \\ 0.95 & 1.00 & \ddots & & 0.95 & \vdots & & & & \vdots \\ \vdots & \ddots & \ddots & \ddots & \vdots & \vdots & & & & \vdots \\ 0.95 & & \ddots & 1.00 & 0.95 & \vdots & & & & \vdots \\ 0.95 & 0.95 & \dots & 0.95 & 1.00 & 0 & \dots & \dots & \dots & 0 \\ 0 & \dots & \dots & \dots & 0 & 1.00 & 0 & \dots & 0 & 0 \\ \vdots & & & & \vdots & 0 & 1.00 & \ddots & & 0 \\ \vdots & & & & \vdots & \vdots & \ddots & \ddots & \ddots & \vdots \\ \vdots & & & & \vdots & 0 & & \ddots & 1.00 & 0 \\ 0 & \dots & \dots & \dots & 0 & 0 & 0 & \dots & 0 & 1.00 \end{bmatrix}$$

Once adding the variability of the outcome Y, the observed values have a correlation of 0.475 between the first 15 pipelines.

Scenario 2: 6 independent pipelines were considered with variance ranging from 0.25 to 15:

$$\Sigma_{\text{scenario 2}} = \begin{bmatrix} 2.5 & 0 & 0 & 0 & 0 & 0 \\ 0 & 0.25 & 0 & 0 & 0 & 0 \\ 0 & 0 & 5.00 & 0 & 0 & 0 \\ 0 & 0 & 0 & 7.50 & 0 & 0 \\ 0 & 0 & 0 & 0 & 10.0 & 0 \\ 0 & 0 & 0 & 0 & 0 & 15.0 \end{bmatrix}$$

After adding the variability of the outcome Y, the standard deviation of the observed outcome for each pipeline relative to the pipeline with the lower variability was respectively 1.67 1.0, 2.19, 2.61, 2.97, 3.58.

Scenario 3: 20 pipelines were considered. 15 of the 20 pipelines were equally highly correlated (correlation of 0.95) and independent of the remaining 5 pipelines, themself independent of each other (correlation of 0). The first 15 pipelines had variance 2.5, and the remaining 0.25, 5, 7.5, 10, 15. The corresponding covariance matrix is displayed below:

$$\Sigma_{\text{scenario 3}} = \begin{bmatrix} \Sigma_{15} & O \\ O & D_5 \end{bmatrix} = \begin{bmatrix} 2.500 & 2.375 & \cdots & 2.375 & 2.375 & 0 & \cdots & \cdots & \cdots & 0 \\ 2.375 & 2.500 & \ddots & & 2.375 & \vdots & & & & \vdots \\ \vdots & \ddots & \ddots & \ddots & \vdots & \vdots & & & & \vdots \\ 2.375 & & \ddots & 2.500 & 2.375 & \vdots & & & & \vdots \\ 2.375 & 2.375 & \cdots & 2.375 & 2.500 & 0 & \cdots & \cdots & \cdots & 0 \\ 0 & \cdots & \cdots & \cdots & 0 & 0.250 & 0 & \cdots & 0 & 0 \\ \vdots & & & & \vdots & 0 & 5.000 & \ddots & & 0 \\ \vdots & & & & \vdots & \vdots & \ddots & \ddots & \ddots & \vdots \\ \vdots & & & & \vdots & 0 & & \ddots & 10.00 & 0 \\ 0 & \cdots & \cdots & \cdots & 0 & 0 & 0 & \cdots & 0 & 15.00 \end{bmatrix}$$

Scenario 4: same as scenario 3 but where the noise distribution is a multivariate Student's t-distribution with 3 degrees of freedom.

Scenario 5: same as scenario 3 but where the noise distribution is the absolute value of a multivariate Gaussian distribution. Taking the absolute value will affect not only the shape of the distribution but also its moments (i.e. mean, variance, and correlation). To retrieve the moments of scenario 3 after applying the absolute value, we simulated using the same multivariate Gaussian distribution as in scenario 3 except that the correlation between the first 15 pipelines was 0.980 instead of 0.95. Then we took the absolute value, subtracted $\frac{\sigma\sqrt{2}}{\sqrt{\pi}}$ and subsequently dividing by $\sqrt{1-\frac{2}{\pi}}$. It can be shown that this procedure will lead to 0-mean noise with variance-covariance matrix $\Sigma_{\text{scenario 3}}$ using the following result: for two jointly normally distributed variables X and Y with mean 0, variance σ^2 and correlation ρ

- $\mathbb{E}[|X|] = \frac{\sigma\sqrt{2}}{\sqrt{\pi}}$
- $\mathbb{V}ar[|X|] = \sigma^2\left(1 - \frac{2}{\pi}\right)$

- $\mathbb{C}or(|X|,|Y|) = \frac{\mathbb{E}[|X||Y|]-\mathbb{E}[|X|]\mathbb{E}[|Y|]}{\sqrt{\mathbb{V}ar[|X|]\mathbb{V}ar[|X|]}} = 2\left(\rho \arcsin(\rho) + \sqrt{1-\rho^2} - 1\right)/(\pi - 2)$
  which follows from $\mathbb{E}[|X||Y|] = \frac{2}{\pi}\left(\rho \arcsin(\rho) + \sqrt{1-\rho^2}\right)\sigma^2$, e.g. see Li and Wei, 2009.

Once adding the variability of the outcome Y, the observed values have a correlation of 0.964 between the first 15 pipelines. Figure A display the density of the distribution of the noise and of the observed values for selected pipelines and scenario.

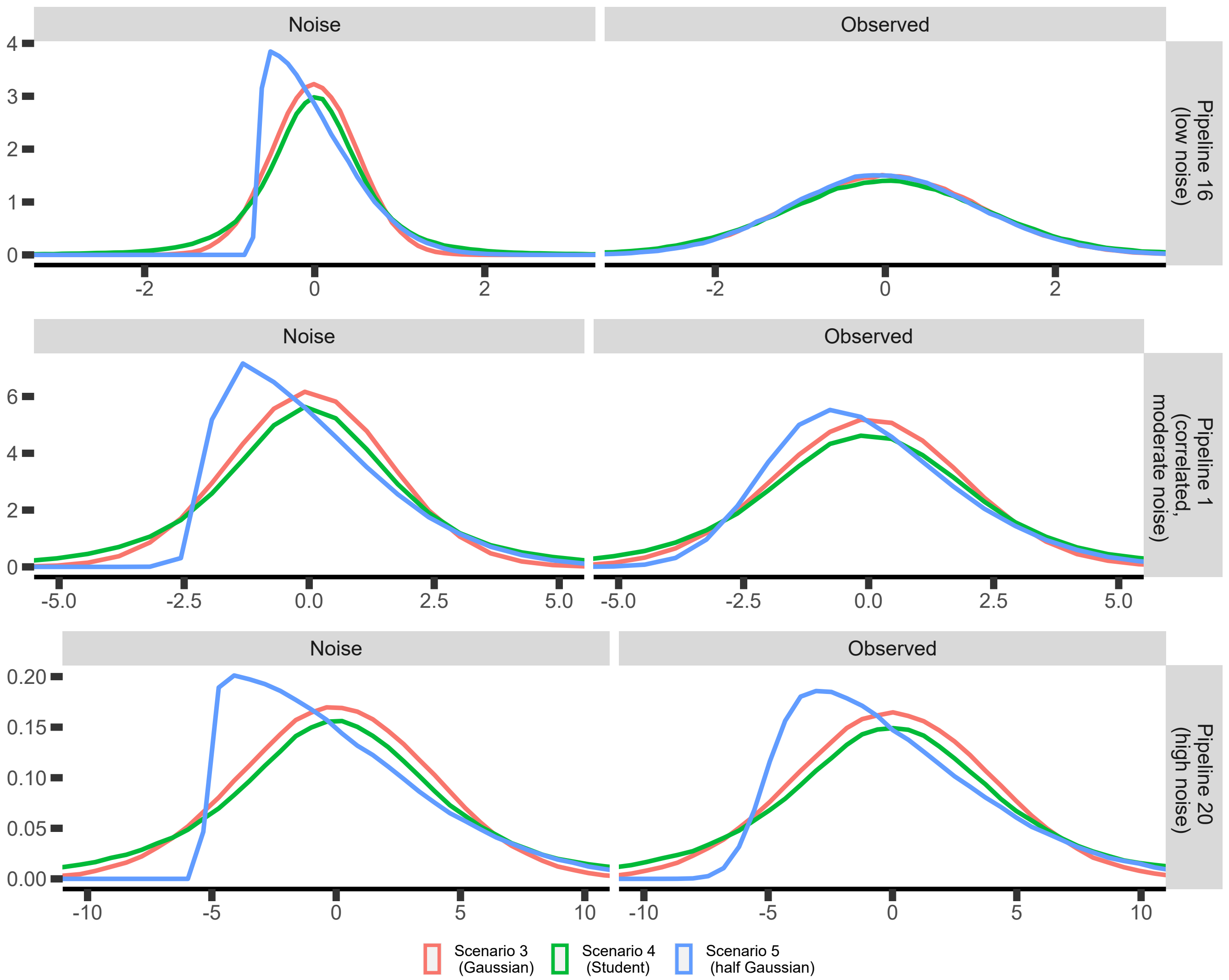

Figure A: Density of the (marginal) distribution of the noise (left panels) and of the observed values (right panels) for three pipelines. Colors refer to different scenarios, i.e., different noise distributions.

B: Influence function

In the univariate case (i.e. $R = P = 1$) without covariate ($C = 0$), the effect of interest ψ may be the Pearson's correlation coefficient:

$$\psi = \frac{\mathbb{E}[XY] - \mathbb{E}[X]\mathbb{E}[Y]}{\sqrt{\mathbb{E}[X^2] - E[X]^2}\sqrt{\mathbb{E}[Y^2] - \mathbb{E}[Y]^2}}$$

when X is continuous or the mean difference when X is binary.

$$\psi = \mathbb{E}[Y|X=1] - \mathbb{E}[Y|X=0]$$

Here $\mathbb{E}[.]$ denotes the expectation and $\mathbb{E}[.|.]$ the conditional expectation. The later case leads to simple expression: $\widehat{\psi}_j$ is the empirical mean difference between the groups

$$\widehat{\psi}_j = \frac{1}{n}\sum_{i=1}^{n}\left(\frac{y_{ij}x_i}{\pi} - \frac{y_{ij}(1-x_i)}{1-\pi}\right)$$

where π denotes the proportion of observations with $X = 1$, y_{ij} the brain signal for individual i processed by pipeline j, and x_i the exposure value for individual i. The previous expression is equivalent to:

$$\sqrt{n}\left(\widehat{\psi}_j - \psi_j\right) = \frac{1}{\sqrt{n}}\sum_{i=1}^{n}\varphi_{\widehat{\psi}_j}(\mathcal{O}_i)$$

where $\varphi_{\widehat{\psi}_j}(\mathcal{O}_i) = \frac{y_{ij}x_i}{\pi} - \frac{y_{ij}(1-x_i)}{1-\pi} - \psi_j$, $\mathcal{O}_j = (y_{i1}, \dots, y_{iJ}, x_i)$, and ψ_j is the large sample value of $\widehat{\psi}_j$. Denoting Y_j the random variable representing the brain signal processed by pipeline j, the estimator can be seen, as the empirical average of independent realizations of a new random variable $\frac{Y_jX}{\pi} - \frac{Y_j(1-X)}{1-\pi}$ and the influence function of $\widehat{\psi}_j$ equals this random variable minus its expected value ψ_j. Thus from the multivariate central limit theorem we get that the joint distribution of the estimates is asymptotically multivariate normal. It has mean ψ (under our assumption that $\forall j \in \{1, \dots, J\}, \psi = \psi_j$) and its variance-covariance, denoted $\Sigma_{\widehat{\psi}}$, is the same as the one of $\boldsymbol{\varphi}_{\widehat{\boldsymbol{\psi}}} = \left(\varphi_{\widehat{\psi}_1}, \dots, \varphi_{\widehat{\psi}_J}\right)$ divided by n. Because $\boldsymbol{\varphi}_{\widehat{\boldsymbol{\psi}}}$ involves some unknown parameters like π and ψ_j we do not observe it and cannot directly estimate $\Sigma_{\widehat{\psi}}$. However, by plugging our estimates of these unknown parameters we can approximate $\varphi_{\widehat{\psi}_j}$ as $\widehat{\varphi}_{\widehat{\psi}_j}(\mathcal{O}_{ij}) = \frac{y_{ij}x_i}{\frac{1}{n}\sum_{i=1}^{n}x_i} - \frac{y_{ij}(1-x_i)}{1-\frac{1}{n}\sum_{i=1}^{n}x_i} - \frac{1}{n}\sum_{i=1}^{n}\left(\frac{y_{ij}x_i}{\pi} - \frac{y_{ij}(1-x_i)}{1-\pi}\right)$ and approximate $\Sigma_{\widehat{\psi}}$.

In a more general case, we would define a statistical model $\mathcal{M}(\Theta)$ relating $\boldsymbol{X}$ and $\boldsymbol{Y}$ via a parameter ψ. ψ may be an element of Θ, the set of model parameters, or a function of elements of Θ. For instance one could use a latent variable model (LVM) with two latent variables, one summarizing the

brain measurements and another summarizing the exposure variables. ψ is then the coefficient relating the two latent variables. See Figure 1 of Stenbæk et al., 2017 for a graphical representation of a LVM - in this example the latent variable "LVu" represents the PET measurement and the latent variable "LVpos" the memory relative to positive word. In this more general case the previous decomposition does not hold exactly but up to a residual term $o_p(1)$ which converges to 0 as the sample approaches infinity:

$$\sqrt{n}\left(\widehat{\psi}_j - \psi_j\right) = \frac{1}{\sqrt{n}}\sum_{i=1}^{n}\varphi_{\widehat{\psi}_j}(\mathcal{O}_i) + o_p(1)$$

This decomposition exists for any estimator $\widehat{\psi}$ derived from an M-estimator (Van der Vaart, 2000, section 5.3), including likelihood-based estimators. Denote by $\widehat{\theta}_j$ the ML estimator and $\widehat{\Psi}_j = c^\intercal\widehat{\theta}_j$ the parameter of interest (c may be a vector starting by 1 and followed by 0's, i.e. selects the first element of $\widehat{\theta}_j$). The corresponding influence function only involves the first two derivatives of the log-likelihood (Tsiatis, 2006, formula 3.6):

$$\varphi_{\widehat{\psi}_j}(\mathcal{O}_i) = -c^\intercal\mathbb{E}\left[\frac{\partial\mathcal{S}_j(\mathcal{O}_i,\theta_j)}{\partial\theta_j}\right]^{-1}\mathcal{S}_j(\mathcal{O}_i,\theta_j)$$

with $\mathcal{S}_j(\mathcal{O}_i,\theta_j)$ being the score for individual i when considering pipeline j, i.e. vector containing the first derivatives of the log-likelihood contribution of individual i. Once the influence function has been estimated for each individual, we can use it to obtain a consistent estimator of $\Sigma_{\widehat{\boldsymbol{\psi}}}$:

$$\widehat{\Sigma}_{\widehat{\boldsymbol{\psi}}} = \frac{1}{n}\sum_{i=1}^{n}\boldsymbol{\varphi}_{\widehat{\boldsymbol{\psi}}}(\mathcal{O}_i)^\intercal\boldsymbol{\varphi}_{\widehat{\boldsymbol{\psi}}}(\mathcal{O}_i)$$

where $\boldsymbol{\varphi}_{\widehat{\boldsymbol{\psi}}}(\mathcal{O}_i)^\intercal$ denotes the transpose of the J-dimensional vector of influence functions relative to individual i. Practically speaking the above allows us assess the variance-covariance matrix of effects across pipelines, e.g., if pipelines are completely independent in terms of their estimated effect, then $\widehat{\Sigma}_{\widehat{\boldsymbol{\psi}}}$ would be a diagonal matrix with the uncertainty of the estimated effect per pipeline in the diagonal. However, if the estimated effects across pipelines are correlated, the matrix would not be sparse.

C: Integration of the Gaussian density - bivariate case

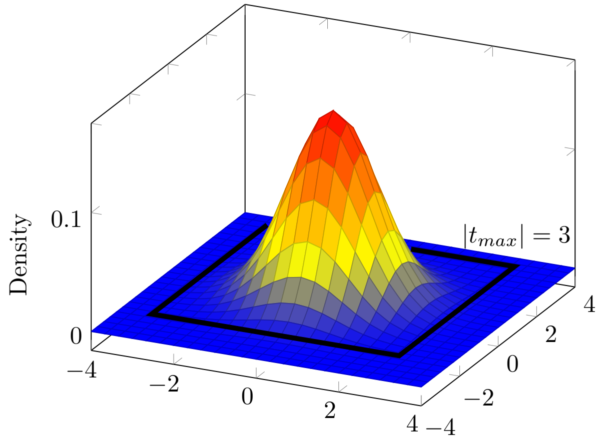

Figure B: Density of the two dimensional standard normal distribution (colored surface). The black line delimits the domain $\mathcal{D}(\widehat{\boldsymbol{t}}_{\max})$ where $J = 2$, $\widehat{t}_1 = 1.5$, $\widehat{t}_2 = 3$, and $\Sigma_{\widehat{\boldsymbol{\psi}}}$ is the identity matrix with two rows and two columns. The area under the blue surface external to the black line corresponds to the p-value relative to the first test, adjusted for two tests.

D: Reformulation of the GLS estimator

D:.1 Via a spectral decomposition

$\widehat{\Sigma}_{\widehat{\psi}}$ being a symmetric semi-positive definite matrix, it admits the decomposition $\widehat{\Sigma}_{\widehat{\psi}} = QDQ^\intercal$ where Q is an orthogonal matrix (i.e. $QQ^\intercal = I_J$, the identity matrix) and D is a diagonal matrix with non negative values $(\lambda_1, \dots, \lambda_J)$. Thus:

$$\widehat{\boldsymbol{\psi}}_{\mathsf{GLS}} = \left(\mathbf{1}^\intercal \widehat{\Sigma}_{\widehat{\Psi}}^{-1} \mathbf{1}\right)^{-1} \mathbf{1}^\intercal \widehat{\Sigma}_{\widehat{\Psi}}^{-1} \widehat{\boldsymbol{\psi}} = \left(\mathbf{1}^\intercal Q D^{-1} Q^\intercal \mathbf{1}\right)^{-1} \mathbf{1}^\intercal Q D^{-1} Q^\intercal \widehat{\boldsymbol{\psi}}$$

We first note that $\bar{Q} = \mathbf{1}^\intercal Q$ is a vector with k-th element equal to the sum (column-wise) of the eigenvectors: $\bar{q}_k = \sum_{j=1}^J q_{kj}$. Then:

$$\mathbf{1}^\intercal Q D^{-1} Q^\intercal \mathbf{1} = \sum_{k=1}^J \bar{q}_k \lambda_k^{-1} \bar{q}_k = \sum_{k=1}^J w_k$$

where $w_k = \bar{q}_k^2 / \lambda_k$. Moreover $\mathbf{1}^\intercal Q D^{-1} Q^\intercal$ is a vector with j-th elements $\sum_{k=1}^J \bar{q}_k \lambda_k^{-1} q_{kj} = \sum_{k=1}^J w_k q_{kj}^*$ where $q_{kj}^* = q_{kj} / \bar{q}_k$. Therefore:

$$\widehat{\Psi}_{\mathsf{GLS}} = \frac{1}{\sum_{k=1}^J w_k} \sum_{k=1}^J w_k \sum_{j=1}^J {q_{kj}^*}^\intercal \widehat{\boldsymbol{\psi}}_j = \sum_{j=1}^J w_j^{GLS} \widehat{\psi}_j$$

where $w_j^{GLS} = \frac{1}{\sum_{k=1}^J w_k} \sum_{k=1}^J w_k {q_{kj}^*}^\intercal$. These weights sum up to 1 since:

$$\sum_{j=1}^n w_j^{GLS} = w^{GLS} \mathbf{1} = \left(\mathbf{1}^\intercal Q D^{-1} Q^\intercal \mathbf{1}\right)^{-1} \mathbf{1}^\intercal Q D^{-1} Q^\intercal \mathbf{1} = 1$$

D:.2 Via joint modeling

Consider the simple case of a single continuous brain measurement ($R = 1$), a single binary exposure ($P = 1$) with probability π of being 1, no covariate ($C = 0$), and no missing value. To simplify the derivations we will only consider the case $\pi = 1/2$. We can use a joint linear model:

$$Y_{ij} = \alpha_j + \beta X_i + \varepsilon_{ij} \text{ where } (\varepsilon_{i1}, \dots, \varepsilon_{iJ}) \sim \mathcal{N}(0, \Sigma_\varepsilon)$$

Denote by $\widehat{\boldsymbol{\mu}}_g = (\widehat{\mu}_{g1}, \dots, \widehat{\mu}_{gJ})$ the vector empirical mean in each group (i.e. one relative to $X = 1$

another to $X = 0$) of sample size $\frac{n}{2}$. Since the mean and variance are sufficient statistics in Gaussian models, the joint linear model is equivalent to:

$$\widehat{\mu}_{gj} = \alpha_j + \beta X_g + e_{gj} \text{ where } (e_{g1}, \ldots, e_{gJ}) \sim \mathcal{N}(0, 2\Sigma_\varepsilon/n)$$

Denote by $\Delta\widehat{\boldsymbol{\mu}} = (\Delta\widehat{\mu}_1, \ldots, \Delta\widehat{\mu}_J)$ the vector of difference in mean, the previous model implies:

$$\Delta\widehat{\mu}_j = \beta + \epsilon_j \text{ where } (\epsilon_1, \ldots, \epsilon_J) \sim \mathcal{N}(0, 4\Sigma_\varepsilon/n)$$

whose maximum likelihood solution is $\widehat{\beta} = \left(\mathbf{1}^\intercal\widehat{\Sigma}_\epsilon^{-1}\mathbf{1}\right)^{-1}\mathbf{1}^\intercal\widehat{\Sigma}_\epsilon^{-1}\Delta\widehat{\boldsymbol{\mu}}$.

Now consider the GLS estimator of the common exposure effect $\widehat{\boldsymbol{\psi}}_{\mathsf{GLS}} = \left(\mathbf{1}^\intercal\widehat{\Sigma}_{\widehat{\Psi}}^{-1}\mathbf{1}\right)^{-1}\mathbf{1}^\intercal\widehat{\Sigma}_{\widehat{\Psi}}^{-1}\widehat{\boldsymbol{\psi}}$ based on the pipeline specific Ordinary Least Squares (OLS) estimators of the exposure effect $\widehat{\boldsymbol{\psi}} = \left(\widehat{\psi}_1, \ldots, \widehat{\psi}_J\right)$. Denote by $Y_{.j}$ the brain measurements across individuals relative to the j-th pipeline, $\Theta_j = (\alpha_j, \psi_j)$ the mean parameters of each pipeline specific model, $Z = (\mathbf{1}, X)$ the corresponding design matrix, and σ_j^2 the residual variance parameter. We have $Z^\intercal Z = \begin{bmatrix} n & n/2 \\ n/2 & n/2 \end{bmatrix}$ whose inverse is $\begin{bmatrix} 2/n & -2/n \\ -2/n & 4/n \end{bmatrix}$. Hence the second element of $Z_i(Z^\intercal Z)^{-1}$ is either $2/n$ or $-2/n$ and $Z_i(Z^\intercal Z)^{-1}cc^\intercal(Z^\intercal Z)^{-1}Z_i{}^\intercal = 4/n^2$. It follows that:

- $\widehat{\psi}_j = (Z^\intercal Z)^{-1}Z^\intercal Y_{.j} = \frac{n}{2}\sum_{i=1}^{n/2} Y_{ij} - \frac{n}{2}\sum_{i=n/2+1}^{n} Y_{ij} = \Delta\widehat{\mu}_j$, assuming that the observations are sorted by group.

- $\varphi_{\widehat{\psi}_j}(\mathcal{O}_i) = -c^\intercal (Z^\intercal Z)^{-1} Z_i^\intercal (Y_{ij} - Z_i\Theta_j) = \pm\frac{2}{n}(Y_{ij} - Z_i\Theta_j)$ since the score relative to $\widehat{\psi}_j$ is $\frac{1}{\sigma_j^2}Z_i^\intercal (Y_{ij} - Z_i\Theta_j)\, c$ and the variance covariance $\sigma_j^2 (Z^\intercal Z)^{-1}$.

- $\widehat{\Sigma}_{\widehat{\psi}} = \frac{1}{n}\sum_{i=1}^{n}\varphi_{\widehat{\boldsymbol{\psi}}}(\mathcal{O}_i)^\intercal\varphi_{\widehat{\boldsymbol{\psi}}}(\mathcal{O}_i) = \frac{4}{n^2}\sum_{i=1}^{n}(Y_{i.} - Z_i\Theta)^\intercal(Y_{i.} - Z_i\Theta) = \frac{4}{n}\widehat{\Sigma}_\varepsilon$

So $\widehat{\boldsymbol{\psi}}_{\mathsf{GLS}} = \left(\mathbf{1}^\intercal\widehat{\Sigma}_\varepsilon^{-1}\mathbf{1}\right)^{-1}\mathbf{1}^\intercal\widehat{\Sigma}_\varepsilon^{-1}\Delta\widehat{\boldsymbol{\mu}}$. If the common effect models holds (i.e. $\widehat{\psi}_1 = \ldots = \widehat{\psi}_J = \beta$) then $\widehat{\Sigma}_\varepsilon$ is an unbiased estimate of Σ_ε so $\widehat{\boldsymbol{\psi}}_{\mathsf{GLS}}$ is asymptotically equivalent to the ML estimator $\widehat{\beta}$ and thus asymptotically efficient.

D:.3 Constrained GLS estimator

Denoting $w_{\max}^{GLS} = \max_{j\in\{1,\dots,J\}} |w_j^{GLS}|$ and $\xi \in \{-1, 1\}$. We find κ by solving:

$$\begin{aligned}
\xi &= \frac{\xi w_{\max}^{GLS}}{\kappa + w_{\max}^{GLS}} + \frac{1}{J}\left(1 - \frac{1}{\kappa + w_{\max}^{GLS}}\right) \\
\xi(\kappa + w_{\max}^{GLS})J &= \xi w_{\max}^{GLS} J + \kappa + w_{\max}^{GLS} - 1 \\
\xi\kappa J &= \kappa + w_{\max}^{GLS} - 1 \\
\kappa &= \frac{1 - w_{\max}^{GLS}}{1 - \xi J}
\end{aligned}$$

Which admits a unique solution unless the largest GLS weight in absolute value is not unique and can take opposite signs (which is unlikely not further considered). One can then show that the weights sum to 1:

$$\sum_{j=1}^{n} \frac{w_j^{GLS}}{\kappa + w_{\max}^{GLS}} + \frac{1}{J}\left(1 - \frac{1}{\kappa + w_{\max}^{GLS}}\right) = \frac{\sum_{j=1}^{n} w_j^{GLS}}{\kappa + w_{\max}^{GLS}} + 1 - \frac{1}{\kappa + w_{\max}^{GLS}} = 1$$

using that the GLS weights sum to 1.

E: Additional simulation results

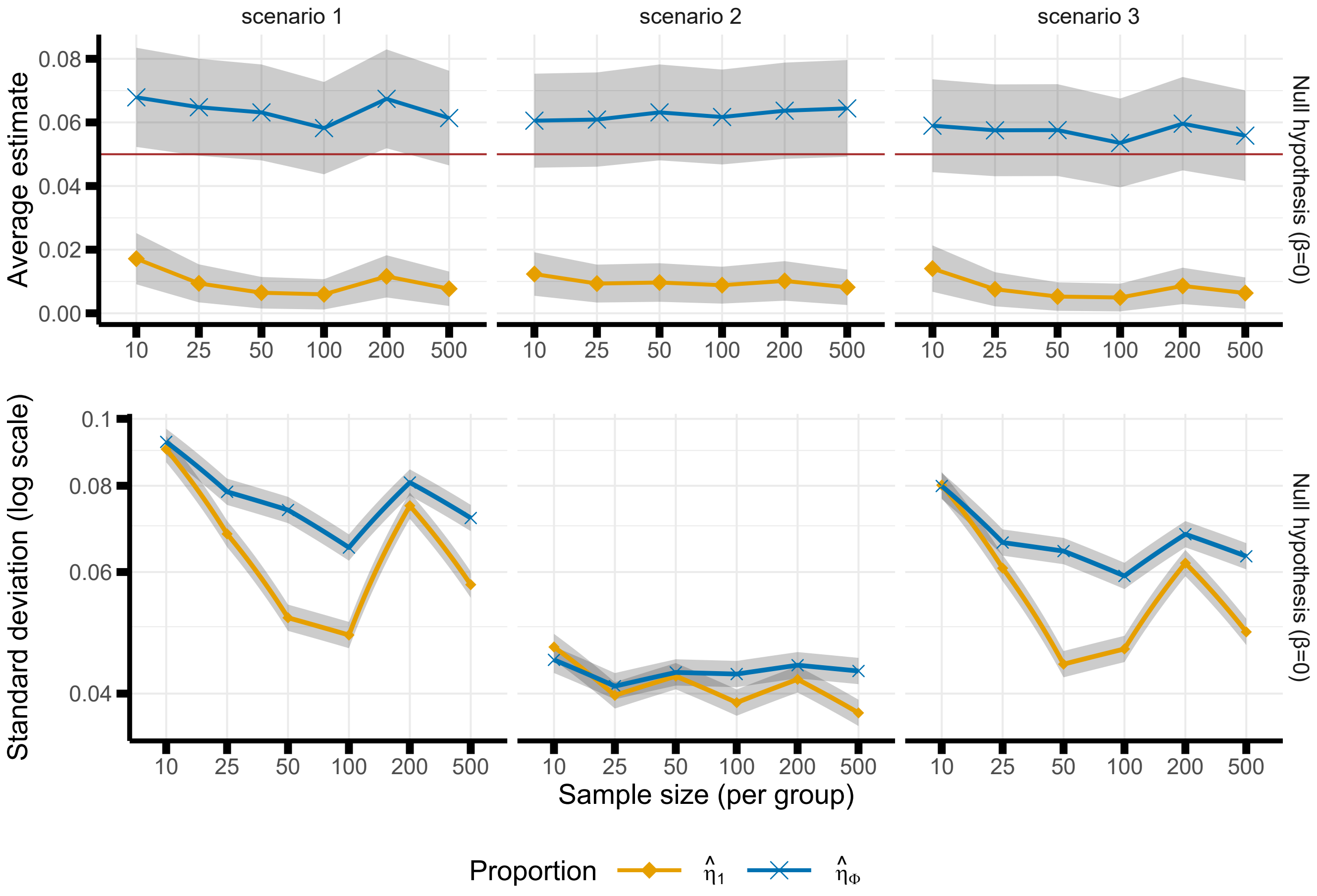

Figure C: Empirical mean (upper panel) and standard deviation (lower panel) of the two proportion estimators ($\widehat{\eta}_{\mathbb{1}}$ and $\widehat{\eta}_{\Phi}$) for each scenario, and sample size under the null hypothesis. Shaded area represents the Monte Carlo uncertainty. The red horizontal line indicates 0.05 which could be considered as a desirable expected value for the proportion of pipelines under the null hypothesis when considering a 5% family-wise error rate.

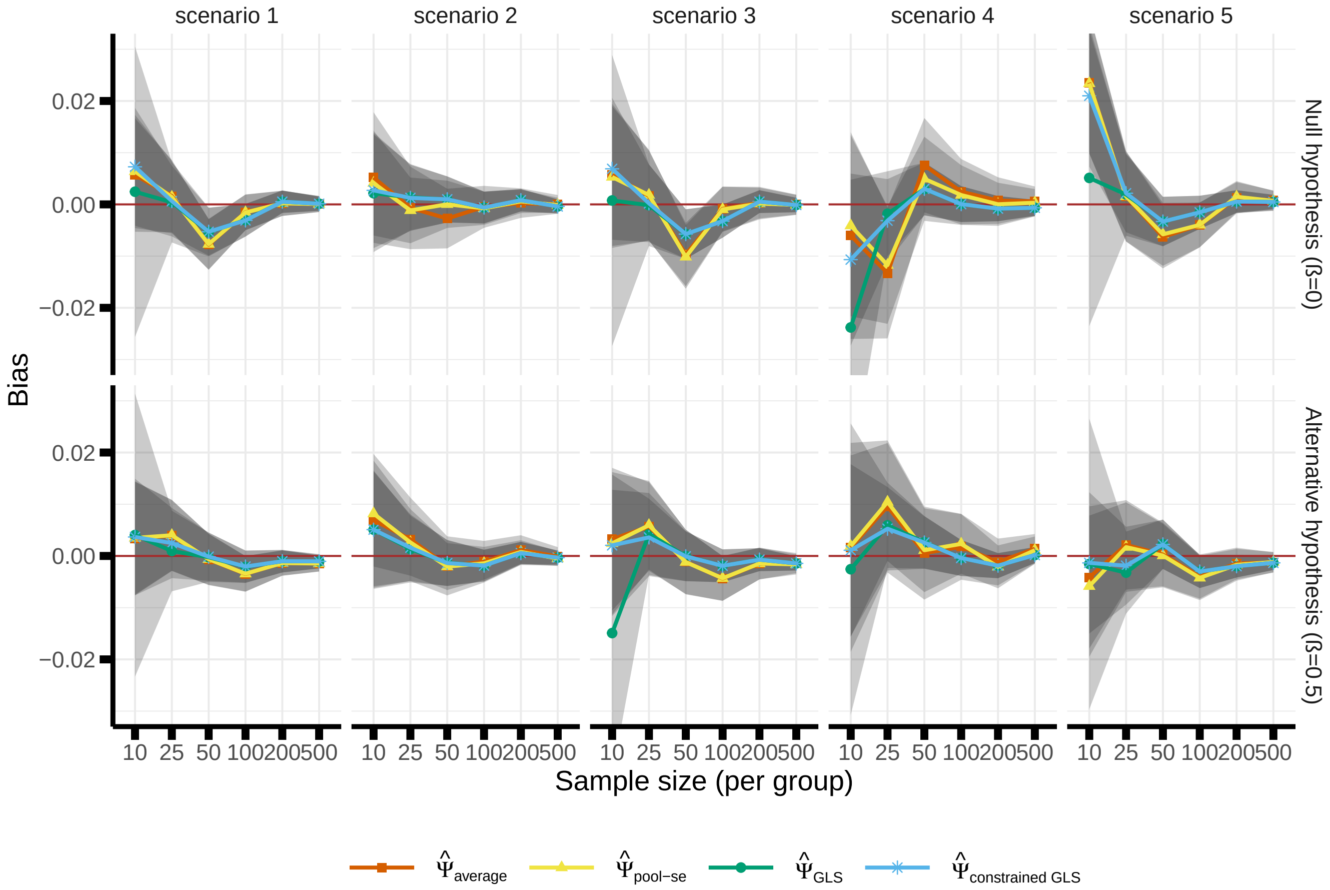

Figure D: Bias of the global effect estimators under the null hypothesis (upper panel) and the alternative hypothesis (lower panel) for each scenario and sample size. Shaded area represents the Monte Carlo uncertainty.

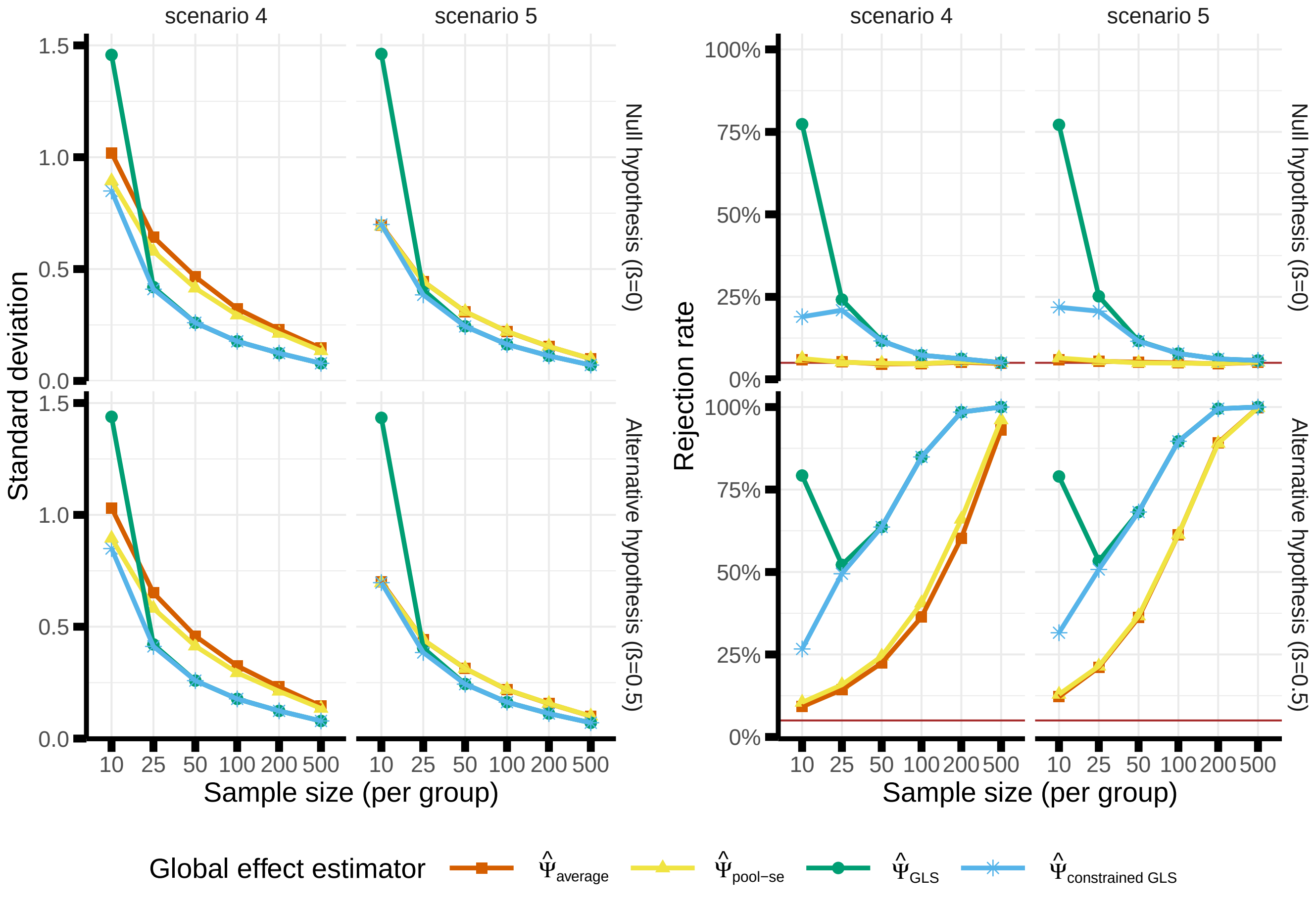

Figure E: Empirical standard deviation and rejection rate of the estimated common effect under non-normally distributed noise.

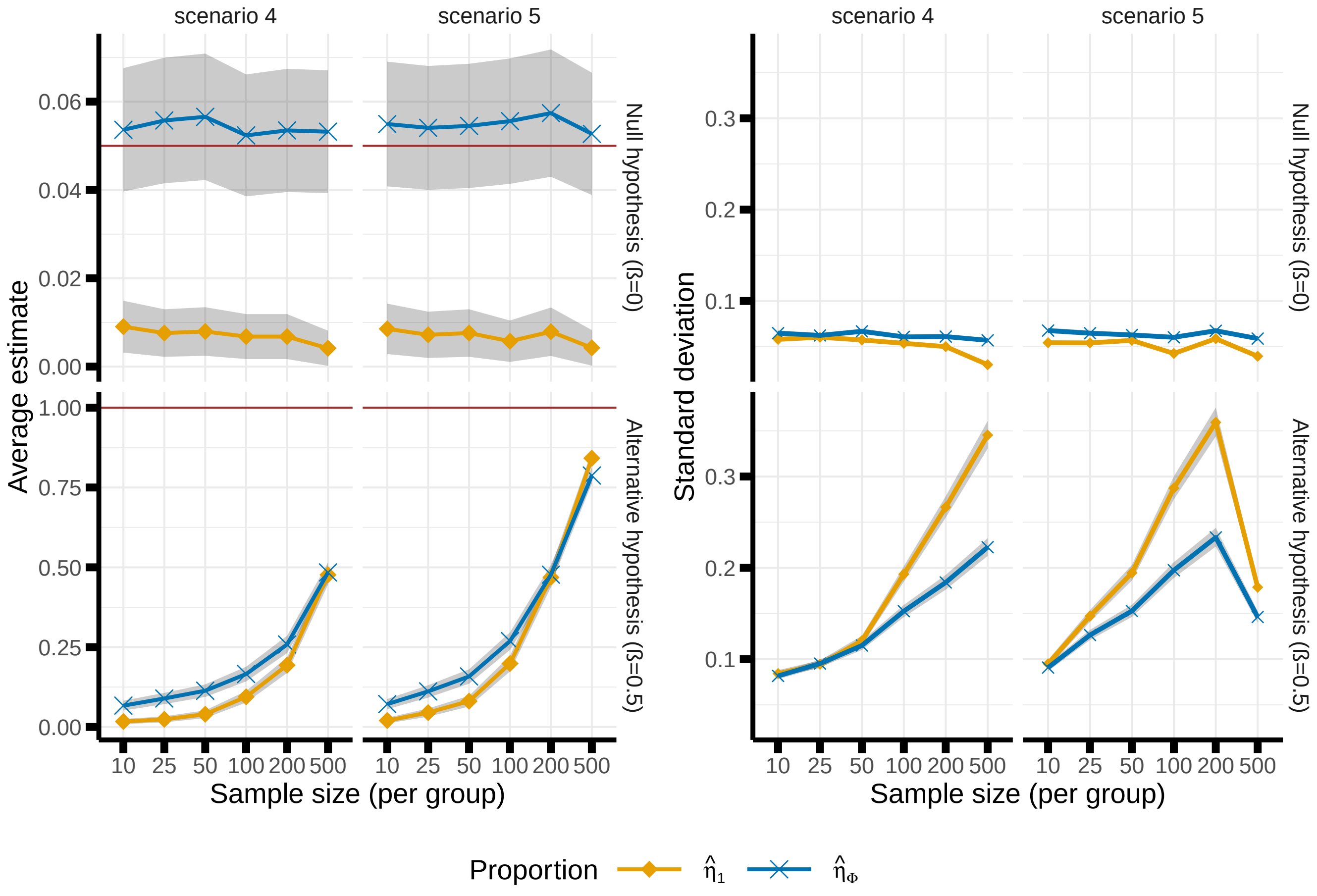

Figure F: Empirical mean and standard deviation of the two proportion estimators ($\widehat{\eta}_{\mathbb{1}}$ and $\widehat{\eta}_{\Phi}$) under non-normally distributed noise. Shaded area represents the Monte Carlo uncertainty. In the top left panel, the red horizontal line indicates 0.05 which could be considered as a desirable expected value for the proportion of pipelines under the null hypothesis when considering a 5% family-wise error rate.

F: Key mathematical notations

Number of:

- P: exposures.
- C: covariates.
- R: measurements per individual (e.g. brain regions).
- n: individuals (i.e. independent observations).
- J: pipelines.

Observed random variables:

- $\boldsymbol{X} = (X_1, \dots, X_P)$: exposures (e.g. treatment).
- $\boldsymbol{W} = (W_1, \dots, W_C)$: covariates (e.g. age, sex).
- $\boldsymbol{Y} = (Y_1, \dots, Y_R)$: outcomes (e.g. brain measurements).
- $(\mathcal{O}_i)_{i \in \{1,\dots,n\}} = (\boldsymbol{y}_i, \boldsymbol{x}_i, \boldsymbol{w}_i)_{i \in \{1,\dots,n\}}$: sample.

Parameters (indexed by j when referring to the estimates for a specific pipeline):

- σ^2 or Σ: variance of the noise for a single or multiple pipelines.
- θ: model parameters, e.g., $\theta = (\alpha, \beta, \sigma^2)$ in a linear regression with intercept α and slope β.
- ψ: effect of interest, can be equal to β (linear regression) or $\exp(\beta)$ (logistic model).
- $\sigma_{\widehat{\psi}}$: standard error of the estimated effect for a single pipeline
  $\Sigma_{\widehat{\psi}}$: variance-covariance matrix of the estimated effect across pipelines.

Random variable used for statistical inference (indexed by j to refer a specific pipeline):

- $\widehat{t} = \frac{\widehat{\psi}}{\sigma_{\widehat{\psi}}}$: Wald test statistic.
- $\varphi_{\widehat{\psi}}$: influence function of the estimated effect.

Global exposure effect (over all pipelines):

- $\widehat{\Psi}_{\text{average}}$: average.
- $\widehat{\Psi}_{\text{pool-se}}$: meta-analysis estimator.
- $\widehat{\Psi}_{\text{GLS}}$: GLS estimator.
- $\widehat{\Psi}_{\text{constrained GLS}}$: constraint GLS estimator.

Proportion of pipelines with evidence for an effect

- $\widehat{\eta}_{\mathbb{1}}$: non-parametric estimator.
- $\widehat{\eta}_{\Phi}$: parametric estimator.

References

Li, W., & Wei, A. (2009). Gaussian integrals involving absolute value functions. *IMS Collections High Dimensional Probability V: The Luminy Volume*, *5*(1), 43–59.

Stenbæk, D. S., Fisher, P. M., Ozenne, B., Andersen, E., Hjordt, L. V., McMahon, B., Hasselbalch, S. G., Frokjaer, V. G., & Knudsen, G. M. (2017). Brain serotonin 4 receptor binding is inversely associated with verbal memory recall. *Brain and behavior*, *7*(4), e00674.

Tsiatis, A. A. (2006). *Semiparametric theory and missing data*. Springer.

Van der Vaart, A. W. (2000). *Asymptotic statistics* (Vol. 3). Cambridge university press.
